# Phosphorylation of the Yeast γ-Tubulin Tub4 Regulates Microtubule Function

**DOI:** 10.1371/journal.pone.0019700

**Published:** 2011-05-05

**Authors:** Tien-chen Lin, Linda Gombos, Annett Neuner, Dominik Sebastian, Jesper V. Olsen, Ajla Hrle, Christian Benda, Elmar Schiebel

**Affiliations:** 1 Zentrum für Molekulare Biologie der Universität Heidelberg, DKFZ-ZMBH Allianz, Heidelberg, Germany; 2 MPI Biochemistry, Martinsried, Germany; Tulane University Health Sciences Center, United States of America

## Abstract

The yeast γ-tubulin Tub4 is assembled with Spc97 and Spc98 into the small Tub4 complex. The Tub4 complex binds via the receptor proteins Spc72 and Spc110 to the spindle pole body (SPB), the functional equivalent of the mammalian centrosome, where the Tub4 complex organizes cytoplasmic and nuclear microtubules. Little is known about the regulation of the Tub4 complex. Here, we isolated the Tub4 complex with the bound receptors from yeast cells. Analysis of the purified Tub4 complex by mass spectrometry identified more than 50 phosphorylation sites in Spc72, Spc97, Spc98, Spc110 and Tub4. To examine the functional relevance of the phosphorylation sites, phospho-mimicking and non-phosphorylatable mutations in Tub4, Spc97 and Spc98 were analyzed. Three phosphorylation sites in Tub4 were found to be critical for Tub4 stability and microtubule organization. One of the sites is highly conserved in γ-tubulins from yeast to human.

## Introduction

Microtubules are dynamic cylindrical polymers involved in numerous cellular functions including intracellular transport, chromosome segregation in mitosis and meiosis, cytokinesis, cell movement and signal sensing. One microtubule typically consists of 13 protofilaments, which are composed of head-to-tail arrays of α-/β-tubulin dimers. This head-to-tail configuration confers microtubules a plus-end and a minus-end with distinctive dynamic properties [1].

γ-tubulin is a member of the tubulin superfamily. In cells, γ-tubulin and the associated γ-tubulin complex proteins (GCPs) are assembled into γ-tubulin complexes. These complexes are localized to the microtubule organizing center (MTOC) such as the mammalian centrosome or the yeast spindle pole body (SPB) and in some organisms also along microtubules [2]. γ-tubulin directly interacts with γ-tubulin at the minus-end of microtubules and promotes de novo assembly of microtubules from tubulin subunits. This initiation of tubulin assembly is called microtubule nucleation [3], [4].

In budding yeast genetic screens identified *SPC97* (spindle pole body component of 97 kDa) and *SPC98* as interacting partners of the γ-tubulin *TUB4* [5], [6], [7]. Spc97, Spc98 and Tub4 constitute the core set of the yeast γ-TuSC (known as the Tub4 complex). The Tub4 complex was later found to be a Y-shaped heterotetramer composed of one molecule of Spc97 and Spc98 and two molecules of Tub4 [8], [9], [10]. Subsequently, homologues of Spc97 and Spc98 were identified in various organisms such as fission yeast, insects, humans and plants [11], [12], [13], [14].

The Tub4 complex is recruited to SPBs through the binding to Spc110 and Spc72, which are the γ-tubulin complex receptors at the nuclear and cytoplamic sides of the SPB, respectively [15]. The Tub4 complex is not localized along the microtubule lattice [16], [17]. So far, besides these two receptors and tubulin, no other proteins have been found to directly interact with the budding yeast Tub4 complex. In contrast, other organisms encode additional subunits that assemble together with the γ-TuSC into the higher order complexes, named large γ-tubulin ring complex (γ-TuRC) [2], [4], [18], [19], [20]. As budding yeast *SPC97* and *SPC98*, fission yeast and *Drosophila* Alp4/GCP2 (the homologue Spc97) and Alp6/GCP3 (the homologue Spc98) were found to be essential for viability. However, subunits of the γ-TuRC are dispensable for viability of cells [14], [21], [22], [23]. This observation suggests that in most organisms the small γ-TuSC is necessary and sufficient for microtubule nucleation, while the additional subunits of γ-TuRC have more specialized functions that are not essential for cell viability.

It has been shown that γ-tubulin complex components, such as other SPB or centrosomal proteins, are phosphorylated in a cell cycle dependent manner [24], [25], [26], [27], [28], [29]. However, only a few post-translational modifications of γ-TuSC subunits and associated proteins have been described and studied on a functional level so far. In mammalian cells, for example, a recent Plk1-dependent phosphoproteome analysis of the early mitotic spindle described phosphorylation sites in GCP2 and GCP3 [30]. The phosphorylation of γ-tubulin on Ser131 by SADB kinase, which regulates centrosome duplication, has also been reported [31]. In budding yeast, Tub4 is phosphorylated at Y445 by an unknown kinase [32], regulating the dynamic behaviour of microtubule plus ends. Spc98, Spc110 and Spc72 are phosphorylated by cell cycle regulating kinases such as cyclin-dependent kinase Cdk1 (Cdc28), Mps1 (mono polar spindle one), and polo-like kinase Cdc5 [24], [25], [29].

To investigate the phospho-regulation of the Tub4 complex, we used mass spectrometry to determine the in vivo phosphorylation sites in the Tub4 complex purified from yeast cells after pGal1-co-expression of *SPC97-TAP*, *SPC98* and *TUB4* [29]. This analysis identified more than 50 phosphorylation sites of the Tub4 complex and its associated receptors Spc72 and Spc110. Mutational analysis revealed three phosphorylation sites in Tub4 that either regulate the stability of the protein (S360) or are important for microtubule function (S74 and S100).

## Results

### Identification of phosphorylation sites in Tub4 complex subunits and the receptors Spc72 and Spc110

**Figure 1:**
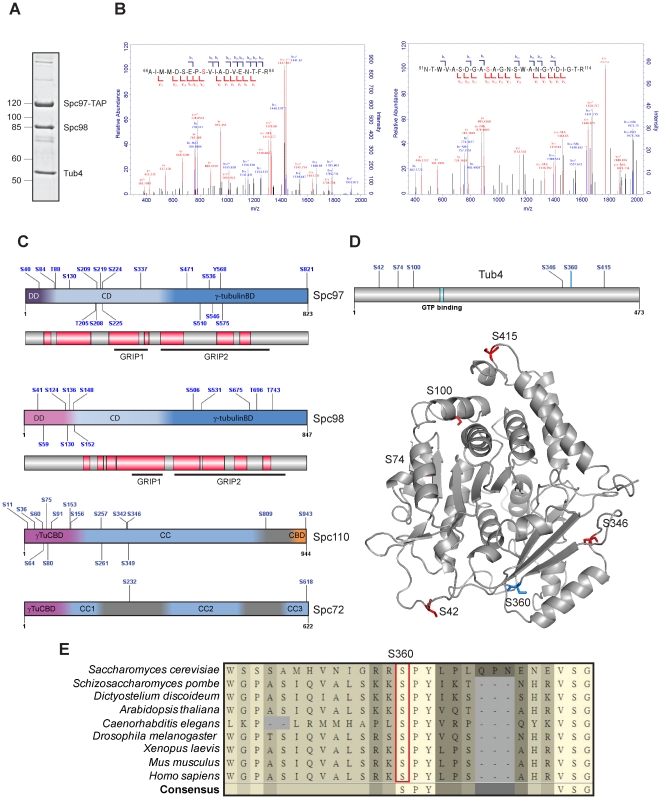
Phosphorylation sites in yeast γ-TuSC. (A) SDS-PAGE analysis of the γ-tubulin complex after Gal1-induced expression of *SPC97-TAP*, *SPC98* and *TUB4*. Spc97-TAP was purified with an IgG-Sepharose column. Coomassie Blue staining visualized the core components of the Tub4 complex. (B) MS/MS spectra of the doubly charged phospho-peptides AIMMDSEPSVIADVENTFR (66–84) and NTWVASDGASAGNSWANGYDIGTR (91–114) from Tub4. The red marked serine residues S74 and S100 were identified to be phosphorylated. b- and y-type ions observed in the MS/MS spectra are shown. (C) Diagram summarising the key domain features of Spc97, Spc98, Spc110 and Spc72 and the distribution of the identified phosphorylation sites. The conserved regions (red blocks) of Spc97 and Spc98 were determined by protein sequence alignment of corresponding orthologous proteins from at least seven species (see Figure 1E). DD: dimerization domain; CD: central domain; γ-tubulinBD: γ-tubulin binding domain; GRIP: gamma ring protein domain; γ-TuCBD: γ-tubulin complex binding domain; CC: coiled-coil domain; CBD: calmodulin binding domain. (D) Phosphorylation sites in Tub4. Top: Position of the identified phosphorylation sites (S42, S74, S199, S346 and S415), the cyclin-dependent kinase (Cdk1) consensus site S360 and the GTP binding site in Tub4. Bottom: The 3D structure was generated by ESyPred3D homology modelling using human γ-tubulin crystal structure “3CB2, chain A” as template. Identified phosphorylation sites are shown in red and the cyclin-dependent kinase (CDK) consensus site in blue. (E) Protein sequence alignment of Tub4 with γ-tubulin from other organisms covering the region around residue S360. S360 and the flanking region on Tub4/γ-tubulin are highly conserved and match the minimal consensus motif of Cdk1 (S/T-P) or of Pho85, a cyclin-dependent kinase (S/T-P-X-Ψ, where Ψ is any hydrophobic residue [72]).

To gain further insights into phospho-regulation of the Tub4 complex, we immunopurified Spc97 fused to the tandem affinity purification (TAP) tag [33] from yeast cells. *SPC97-TAP*, *SPC98* and *TUB4* were co-expressed from the inducible Gal1 promoter by the addition of galactose to the growth medium [29]. TAP tag purification of Spc97-TAP resulted in co-purification of the Tub4 complex subunits Tub4 and Spc98, and of the receptors Spc72 and Spc110 (Figure 1A). After elution from IgG beads with TEV protease the majority of the Tub4 complex migrated by gel filtration as the recombinant Tub4 complex purified from insect cells (E.S. unpublished) [9]. Mass spectrometry analysis of the enriched proteins identified more than 50 phosphorylation sites: 2 in Spc72, 20 in Spc97, 12 in Spc98, 16 in Spc110 and 5 in Tub4 (Figure 1B; Figure S1 and Table S1). Several of the identified phosphorylation sites match consensus motifs for casein kinase 2 (CK2; S/T-X-X-E/D/pT/pS), Cdc5 polo like kinase (I/L/V-E/N/D/Q-X-pS/pT-I/L/V), Cdk1 (pS/pT-P-X-K/R) and Ipl1 aurora B kinase (R/K-X-pT/pS-I/L/V) (Table S1) [34], [35]. Three previously reported phosphorylation sites, two in Tub4 (Y445 and S360) and one in Spc98 (S159), were not detected in our analysis [32], [36], [37]. This could reflect the transient nature of the phosphorylation events or be a consequence of the over-expression of Tub4 complex subunits.

The N-termini of Spc97 and Spc98 interact in the Y shaped Tub4 complex [10] (Figure S2). Interestingly, several phosphorylation sites cluster in the N-terminal dimerization domain of Spc97 and Spc98 (Figure 1C; Spc97: S40, S84, T88 and Spc98; S41 ad S59 and Figure S2). This raises the possibility that interaction between Spc97 and Spc98 is regulated by phosphorylation. Additionally, our analysis identified six phosphorylation sites in the GRIP2 domain of Spc97 (S471 – S575) (Figure 1C). The GRIP motif is present in most γ-TuRC subunits and is presumed to be important for the binding of GCPs to γ-tubulin [4]. Thus, phosphorylation sites in the GRIP domain of Spc97 may regulate the interaction with Tub4.

To evaluate the role of Tub4 phosphorylation, we mapped the phosphorylation sites onto a tertiary structure model of Tub4 that is based on the structure of human γ-tubulin (Figure 1D) [38]. Despite the relatively low sequence similarity to human γ-tubulin (∼35%) [39], this model allowed us to have a view of the location and thus possible function of these phosphorylation sites. The five phosphorylation sites that were identified in this study and S360 [37] are exposed on the surface of the Tub4 molecule. Three phosphorylation sites (S74, S100 and S415) are located at the putative interface between Tub4 and the α-/β-tubulin heterodimer. The other three sites (S42, S346 and S360) may face Spc97 and Spc98 in the Tub4 complex [10], [40]. Therefore, phosphorylation of Tub4 could play a role in the assembly of the Tub4 complex and/or in the interaction between the Tub4 complex and tubulin.

Notably, only one of the phosphorylated residues of Tub4 (Tub4-S360) is highly conserved from yeast to humans (Figure 1E). Tub4-S360 matches the minimal Cdc28/Cdk1 consensus sequence S/T-P. Accordingly, a phosphopeptide encompassing Tub4-S360 has been identified in a proteomic study for Cdc28/Cdk1 substrates [37]. Tub4 has three additional putative Cdc28/Cdk1 sites, T63, S262 and T273, which, however, were not identified in the proteomic study [37]. Because the analysis of phospho-mimicking and non-phosphorylatable mutations of Tub4-T63, -S262, -T273 and -S360 only identified an essential function for Tub4-S360 (see below), we did not analyze Tub4-T63, -S262, -T273 any further.

### Phosphorylation site mutants identify essential phospho-regulated residues in Tub4

To test the functional relevance of the phosphorylation in the Tub4 complex, we systematically mutated the identified phosphorylation sites to phospho-mimicking aspartate or glutamate residues (S/T to D/E) or to non-phosphorylatable alanine residues (S/T to A). Strains expressing the mutated genes (under the control of the endogenous promoters) as the only source of the respective proteins were examined for growth defects using a plasmid shuffle approach [41]. All tested phospho-mimicking and non-phosphorylatable mutants of *SPC97* and *SPC98* were viable at 23°C (Figure S1A). The *spc97-S40A/E*, *spc97-S84E* and *spc97-T88A/E* mutant alleles caused slow growth at all temperatures tested. In contrast, *spc97-S130E* and *spc97-S471E* resulted in a conditional growth defect at 37°C (Figure S1A). Introduction of several phosphorylation site mutations in the region between the dimerization domain and the coiled-coil domain of Spc98 (*spc98-S124E S130E S136E S148E S152E)* did not cause an obvious growth defect (data not shown). Thus, no single phosphorylation site is essential for the function of Spc97 or Spc98.

**Figure 2:**
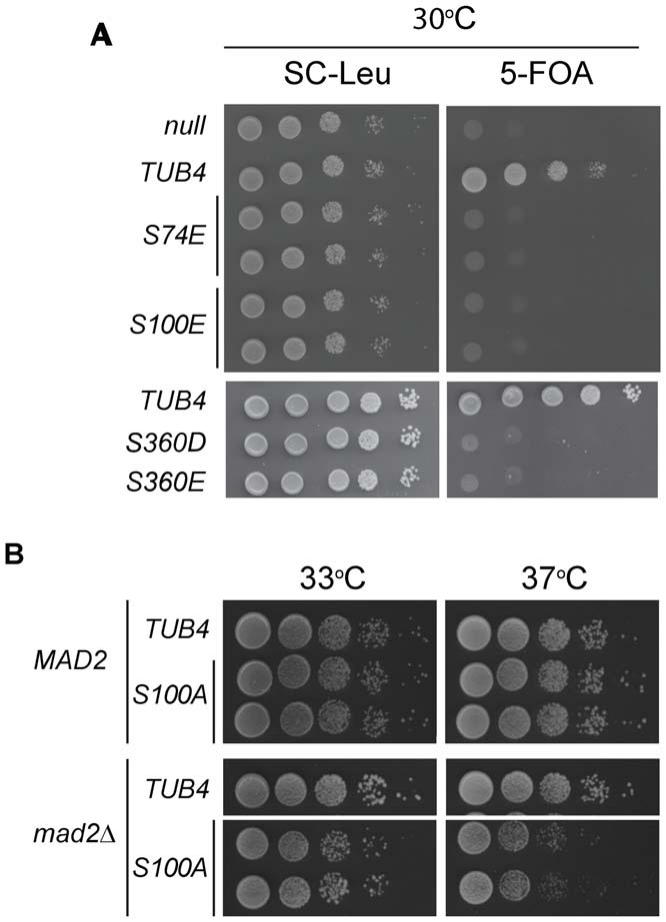
*tub4-S74E*, *tub4-S100E*and*tub4-S360D/E*cells are lethal. (A) Growth assay showing that *tub4-S74E, tub4-S100E* and *tub4-S360D/E* cells are lethal. The *tub4Δ* pRS316-*TUB4* shuffle strain was transformed with *LEU2*-based plasmids (pRS315) containing various *TUB4* alleles. Transformants were grown on SC-Leu or 5-FOA plates for three days at 30°C. 5-FOA only allows cells to grow that have lost the *URA3*-based pRS316-*TUB4*. Thus, in cells that grown on 5-FOA plates, the pRS315 encoded *TUB4* allele is the only source of Tub4 activity. (B) *tub4-S100A* genetically interacts with *mad2Δ*. *tub4-S100A mad2Δ* cells showed a temperature-sensitive growth defect at 37°C.

Analysis of phospho-mimicking and non-phosphorylatable mutants of *TUB4* revealed essential functions for the phosphorylation sites S74, S100 and S360. Cells expressing the phospho-mimicking *tub4-S74E*, *tub4-S100E* or *tub4-S360E/D* mutants, but not the non-phosphorylatable *tub4-S74A*, *tub4-S100A* and *tub4-S360A* mutants, were inviable (Figure 2A and Figure S1B). In contrast, cells with phospho-mimicking or non-phosphorylatable mutations in one of the three putative Cdc28/Cdk1 sites of *TUB4* (T63, S262 and T273) or in *TUB4-S42*, *-S346* or *-S415* did not show growth phenotypes (data not shown). We conclude constitutive phosphorylation of Tub4 at S74, S100 or S360 impairs its essential function.

### *tub4-S100A* shows genetic interaction with the spindle assembly checkpoint gene *MAD2*

Mad2 is part of the spindle assembly checkpoint (SAC), which delays cell cycle progression in response to defective nuclear microtubule-kinetochore interactions, whereas Bub2 is a component of the spindle orientation checkpoint (SPOC) that becomes important when cytoplasmic microtubules are defective [42], [43], [44], [45]. In yeast, *MAD2* and *BUB2* are not essential for viability, but become essential when microtubules are defective [16], [43], [46]. SAC and SPOC then delay cell cycle progression, which allows repair of microtubule defects. Microtubule defects may therefore be masked by the actions of SAC and SPOC.

To test for this possibility, growth of mutant *tub4* cells was analyzed in an otherwise wild type background or in cells deleted in *MAD2* or *BUB2*. *tub4-S100A* cells showed normal growth at all temperatures tested, but showed a strongly reduced growth at 37°C in the *mad2Δ* or *bub2Δ* background cells (Figure 2B and Figure S1B). In contrast, the other non-phosphorylatable or phospho-mimicking (*tub4-S42*, *tub4-S346* or *tub4-S415*) *tub4* mutants did not genetically interact with *MAD2* or *BUB2* (Figure S1B). These data suggest that the *tub4-S100A* mutation causes microtubule defects that are partially compensated by the SAC and SPOC. Consistent with this notion, about 80% of *tub4-S100A mad2Δ* cells had disorganized nuclear microtubules at the restrictive temperature of 37°C, whereas *tub4-S100A* cells had relative normal spindles (Figure S3). Our results suggest that the lack of phosphorylation of Tub4-S100 activates the SAC.

### Constitutive phosphorylation of Tub4 at S74, S100 and S360 interferes microtubule organization

**Figure 3:**
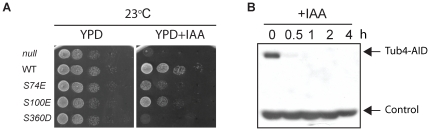
Auxin-induced degradation of Tub4. (A) *TUB4-AID* cells with empty integration vector pRS305 (“null”) or pRS305 with *TUB4* (“WT”), *tub4-S74E*, *tub4-S100E* or *tub4-S360D* were grown on YPD or YPD plates with IAA at 23°C. (B) Western blot analysis of *TUB4-AID* cells with anti-Tub4 antibodies after the addition of IAA (t = 0). The lower molecular weight band (“Control”) is a protein that cross reacts with the anti-Tub4 antibodies and was used as loading control.

Tub4 organizes cytoplasmic and nuclear microtubules at the cytoplasmic and nuclear sides of the SPB, respectively [16], [32]. There is evidence that the two Tub4 complex pools at the SPBs are regulated differently [29]. Thus, phospho-mimicking *TUB4* mutants may impair organization of only one or both microtubule sets. In addition, the nature of the microtubule organization and cell cycle progression defects will give insights into the malfunction of the mutant Tub4 protein. We therefore analyzed cell cycle progression and microtubule organization in *tub4-S74E*, *tub4-S100E* and *tub4-S360D* cells. Because the *tub4* mutants did not support growth of cells, all subsequent experiments were performed in the *TUB4-AID* background (Figure 3A). In these cells endogenous *TUB4* is tagged with the auxin degron [47], allowing for conditional and rapid depletion of Tub4-AID below detectable levels upon cell treatment with indole-3-acetic acid (IAA) (Figure 3B). Accordingly, *TUB4-AID* cells (“null”), but not *TUB4-AID* cells carrying an additional copy of wild type *TUB4* (“WT”), were unable to grow on IAA plates (Figure 3A). *TUB4-AID* cells with *tub4-S74E*, *tub4-S100E* or *tub4-S360D* showed strongly reduced or no growth on IAA plates (Figure 3A). The weak growth of *tub4-S74E* and *tub4-S100E* cells probably arose from the residual function of the mutant Tub4 proteins combined with low levels of Tub4-AID that may remain despite the presence of IAA.

**Figure 4:**
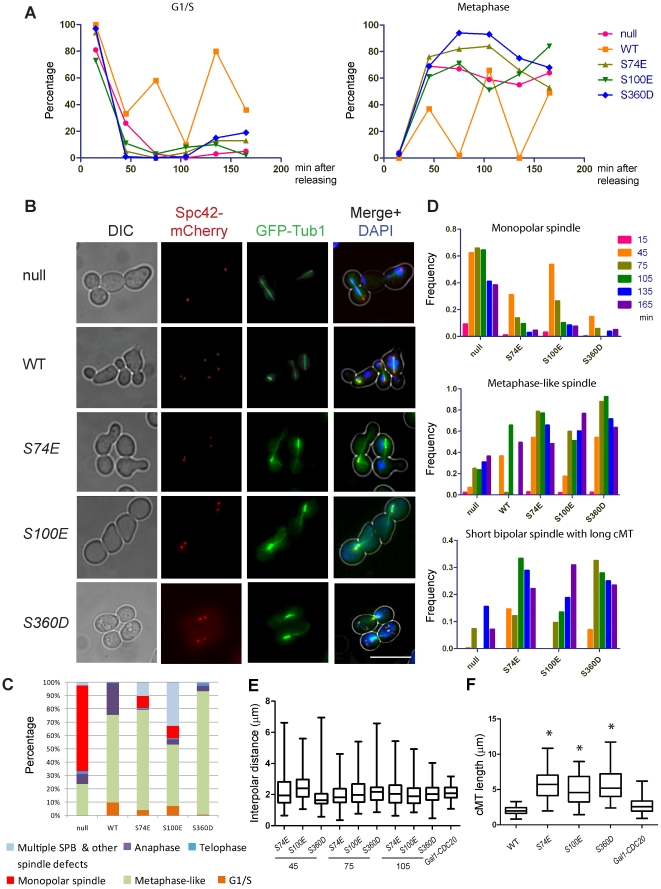
Phospho-mimicking *tub4-S74E*, *tub4-S100E* and *tub4-S360D* mutant cells have abnormal microtubules. (A) *TUB4-AID* cells with empty vector (“null”), *TUB4* (“WT”), *tub4-S74E*, *tub4-S100E* or *tub4-S360D* were arrested in G1-phase with α-factor. IAA was added 30 min before G1 release by washing cells with YPAD containing IAA (G1 release t = 0). Cells were fixed at the indicated time points with paraformaldehyde. DNA was stained with DAPI and cells were analyzed by fluorescence microscopy. *SPC42-mCherry* is a SPB marker; *GFP-TUB1* encodes the yeast α-tubulin gene. N>150 cells per time point were categorized as indicated. (B) Spindle phenotypes of *tub4-S74E*, *tub4-S100E* and *tub4-S360D* cells. Cells of (A) were sampled 75 min after G1 release and analyzed by fluorescence microscopy. Scale bar: 10 µm. (C) Quantification of phenotypes of cells in (B) 75 min after G1 release. N>100 cells per mutant were analyzed as indicated in the figure. (D) Time dependent changes of null, *tub4-S74E*, *tub4-S100E* and *tub4-S360D* cells from (A) with monopolar spindle, metaphase-like spindle and short bipolar spindle and long cMTs. N>150 cells per time point. (E) Length of metaphase-like spindles over time. Metaphase-like cells from (A) were analyzed for spindle length. The spindle length of Gal1-*CDC20* cells was used as reference (N >100 with the exception of *tub4-S100E* cells where N was 41). The significance of the difference between Gal1-*CDC20* cells and mutants at *p*<0.05 was determined by one-way ANOVA. (F) *tub4-S74E, tub4-S100E* and *tub4-S360D* cells with a short bipolar spindle showed longer cytoplasmic microtubules than metaphase arrested Gal1-*CDC20* cells. *TUB4-AID* cells from (A) were sampled 75 min after G1 release. The length of the cytoplasmic microtubules (distance from the SPB to cell cortex) was determined from 3D reconstitution of cells. *tub4-S74E, tub4-S100E* and *tub4-S360D* cells showed longer cytoplasmic microtubules in comparison to wild-type and metaphase-arrested Gal1-*CDC20* cells (n>100). Significance of the difference between wild-type and mutants at *p*<0.05 was determined by one-way ANOVA and is indicated by an asterisk.

For cell cycle analysis, all strains were synchronized in G1 with α-factor. IAA was added 30 min before release from the α-factor block to induce degradation of Tub4-AID. Upon α-factor washout, “WT” cells showed synchronous progression through S phase, metaphase, anaphase and telophase (Figure 4A). “Null” and *tub4-S74E*, *tub4-S100E* or *tub4-S360D* cells progressed through G1/S and arrested in metaphase as large budded cells with unsegregated chromosomes (Figure 4A, B). This phenotype is likely caused by the activation of the spindle assembly checkpoint as a result of defective nuclear microtubules [46]. Accordingly, deletion of *MAD2* abolished the metaphase arrest of the three phospho-mimicking mutants, resulting in an increased population of multi-budded cells (Figure S4A, B), which is an indication for continued cell cycle progression despite microtubule defects [44], [45].

We used the SPB marker Spc42-mCherry and the microtubule marker GFP-Tub1 (yeast α-tubulin) to visualize the microtubule cytoskeleton in synchronized populations [26], [48]. A large fraction of “null” cells arrested with a monopolar spindle containing only one or two juxtaposed SPB signals (Figure 4B–D). In contrast, most *tub4-S74E*, *tub4-S100E* and *tub4-S360D* cells separated their SPBs and assembled short bipolar spindles 75 min after release from G1 (Figure 4C). However, *tub4-S100E* cells were delayed in spindle formation in comparison to *tub4-S74E* or *tub4-S360D* cells. Only 17% of *tub4-S100E* cells assembled a metaphase-like spindle 45 min after release from G1, compared to ∼60% of *tub4-S74E* and *tub4-S360D* cells (Figure 4D, middle panel). Since nuclear microtubules are essential for SPB separation [49], the spindle formation delay suggests a more severe defect in nuclear microtubule organization in *tub4-S100E* cells than in *tub4-S74E* and *tub4-S360D* cells.

Next we measured the length of bipolar spindle and cytoplasmic microtubules, which is an indication of microtubule defects. Because *tub4* mutant cells arrested in metaphase, while wild-type cells continued cell cycle progression, we used pGal1-*CDC20* cells that arrest in metaphase due to lack of activity of the anaphase promoting complex (APC) [50], as reference for metaphase cells without microtubule defects. The average distance between SPBs in *tub4-S74E*, *tub4-S100E* and *tub4-S360D* cells with bipolar spindles remained relatively constant after SPB separation and was similar to the one observed in *CDC20*-depleted cells (Figure 4E). However, the large variation in interpolar distance and the continued metaphase-like cell cycle arrest indicate nuclear microtubule defects in *tub4-S74E*, *-S100E* and *-S360D* cells. In addition, the length of cytoplasmic microtubules was increased in the *tub4-S74E*, *-S100E* and *-S360D* mutant cells compared to wild type metaphase cells (only wild type cells in metaphase with a nuclear spindle of 1.5-2 µm were counted) or *CDC20*-depleted cells (Figure 4F and Figure S5). This suggests changes in cytoplasmic microtubule dynamics in *tub4-S74E*, *-S100E* and *-S360D* mutant cells. Nevertheless, no significant spindle mis-positioning was observed in these *tub4* mutant cells (data not shown). Taken together, we conclude that the *tub4-S74E*, *tub4-S100E* and *tub4-S360D* phospho-mimicking mutations affect the function of both the nuclear and the cytoplasmic microtubules.

### *tub4-S74E, tub4-S100E* or *tub4-S360D* cells show misorganized spindle microtubules

**Figure 5:**
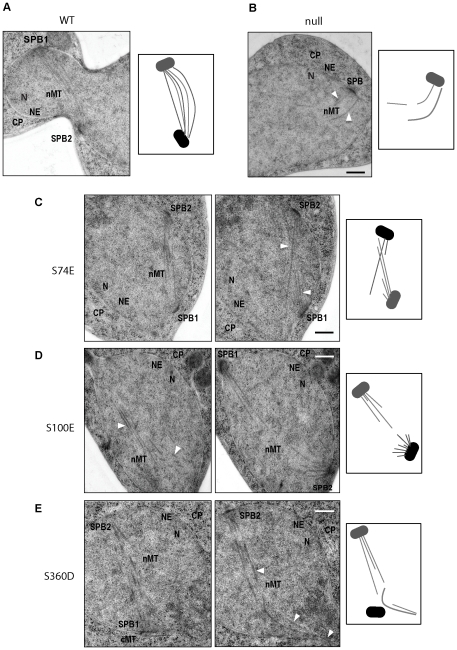
Analysis of the microtubule phenotype of *tub4-S74E*, *tub4-S100E* and *tub4-S360D* cells by thin section electron microscopy. (A-E) *TUB4-AID* cells with the wild type *TUB4* (“WT”; A), empty plasmid (“null”; B), *tub4-S74E* (C), *tub-S100E* (D) or *tub4-S360D* (E) were synchronized with α-factor and treated with IAA as described in Figure 4A. 75 min after the G1 release cells were prepared for thin serial sectioning electron microscopy as described in Materials and Methods. (C–D) Shown are two consecutive sections. The white arrows indicate the position of defective nuclear microtubules. Abbreviations: CP, cytoplasm; cMT, cytoplasmic microtubules; N, nucleus; NE, nuclear envelope; nMT, nuclear microtubules; SPB, spindle pole body. Scale bar: 200 nm.

*tub4-S74E*, *-S100E* or *-S360D* cells were able to assemble a short bipolar-like spindle (Figure 4). However, in many cells the pole to pole distance was shorter than in wild type cells indicating nuclear microtubule defects that we further investigated by thin section electron microscopy (EM). As before, we synchronized *TUB4-AID* cells with α-factor and treated them with IAA to induce Tub4-AID degradation (Figure 5). Large budded “WT” cells assembled bipolar spindles with separated SPBs. Microtubule minus ends were attached to the SPBs and nuclear microtubules were organized in an antiparallel fashion (Figure 5A and Figure S6A). In contrast, mostly one SPB with only a few or no nuclear microtubules were detected in thin sections of *TUB4-AID* “null” cells (Figure 5B and Figure S6B). *tub4-S74E*, *tub4-S100E* and *tub4-S360D* cells showed microtubule organization defects that were distinct from “null” cells. At 45 min or 75 min after release from the G1 block most *tub4-S74E* and *tub4-S360D* cells showed two SPBs separated by 1–2 µm (Figure 5C, E and Figure S6C, E). However, in contrast to wild type cells, nuclear microtubules from opposite poles were often not aligned on the same axis, failing to develop a proper anti-parallel spindle structure (Figure 5C, E; white arrows). Nuclear microtubules of *tub4* cells had a scissor-like appearance (Figure 5C, the 2nd section) or passed one SPB and contacted the nuclear envelope (Figure 5E, the 2nd section). In agreement with fluorescence microscopy analysis (Figure 4D), 45 min after release from the G1 arrest *tub4-S100E* cells showed monopolar spindles with two juxtaposed SPBs (Figure S6D). However, after 75 min we observed mainly bipolar spindles (Figure 5D). As *tub4-S74E* and *tub4-S360D* cells, these *tub4-S100E* cells failed to organize a proper anti-parallel array of nuclear microtubules. Thus, the thin section EM analysis confirmed the nuclear microtubule organization defects in *tub4-S74E*, *tub4-S100E* and *tub4-S360D* cells. The highly abnormal microtubule arrays could be easily discerned without electron tomography analysis and 3D reconstitution of microtubules.

### Tub4 complex formation in *tub4-S74E, tub4-S100E* and *tub4-S360D* cells

**Figure 6:**
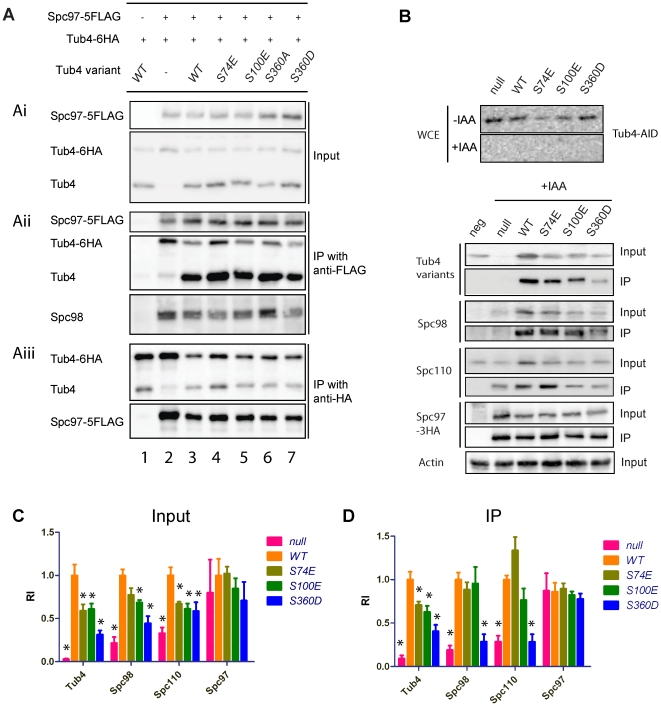
Phospho-mimicking *tub4-S74E*, *tub4-S100E* and*tub4-S360D* alleles affect the interaction between Tub4 complex components and Spc110. (Ai) In the presence of Tub4-6HA cellular levels of the phospho-mimicking Tub4-S74E, Tub4-S100E and Tub4-S360D are similar to Tub4. Lysates from *SPC97 TUB4-6HA* cells (strain YPH499; lane 1), and cells of *SPC97-5FLAG TUB4-6HA* with pRS305 (lane 2), pRS305-*TUB4* (lane 3), pRS305-*tub4-S74E* (lane 4), pRS305-*tub4-S100E* (lane 5), pRS305-*tub4-S360A* (lane 6) and pRS305-*tub4-S360D* (lane 7) were used for anti-FLAG and anti-Tub4 immunoblots. (Aii) Lysates of cells from (Ai) were subjected to immunoprecipitation with anti-FLAG antibodies. Tub4-6HA, Tub4 and Spc98 were analyzed for co-immunoprecipitation using anti-Spc98 and anti-Tub4 antibodies. (Aiii) Anti-HA immunoprecipitations using cells lysates from (Ai). The immunoprecipitations were analyzed with anti-Tub4 and anti-FLAG antibodies. Input is 5% of the immunoprecipitation input. (B) *TUB4-AID SPC97-3HA* cells carrying pRS305 (“null”) or *TUB4* (“WT”), *tub4-S74E*, *tub4-S100E* or *tub4-S360D* on integration vector pRS305 were incubated with IAA for 2 h at 30°C to induce degradation of Tub4-AID. Degradation of Tub4-AID in the presence of IAA was confirmed by immunoblotting (top panel). Lysates of *SPC97* wild type cells incubated in the presence of IAA were subjected to anti-HA IP as negative control (“neg”) to exclude non-specific binding of proteins to beads. The IPs were analyzed by immunoblotting with anti-Tub4, anti-Spc98, anti-Spc110 and anti-HA (Spc97-3HA blot) antibodies. Anti-actin antibodies were used to normalize loading. (C) Tub4-S360D and Spc98 levels were reduced in the absence of wild-type Tub4-AID activity. Input protein levels of cleared cell extracts from (B) were quantified and normalized for actin. The normalized value of wild-type Tub4 cells was set to one. Mean and standard deviation of at least three independent cell extracts are shown. Significance of the difference between wild-type and mutants at *p*<0.05 was determined by one-way ANOVA and is indicated by an asterisk. (D) Phospho-mimicking mutations in Tub4 affect binding to Spc110. Quantified protein levels of Tub4 complex proteins and Spc110 in the Spc97-3HA immunoprecipitations were normalized to precipitated Spc97-3HA. The normalized value of wild-type Tub4 was set as one. Mean and standard deviations of at least three independent samples are shown. Significance of the difference between wild-type and mutants at *p*<0.05 was determined by one-way ANOVA and is indicated by an asterisk. RI: relative intensity.

Tub4 phosphorylation may regulate complex formation with Spc97 and Spc98. This should affect co-immunoprecipitation efficiency between the phospho-mimicking Tub4 mutant proteins, Spc97 and Spc98. The presence of two Tub4 molecules in the Tub4 complex [8], [10], [40] raises the possibility of Tub4 hetero-incorporation. Therefore we first asked how the Tub4 phospho-mimicking mutant proteins assemble into complexes in the presence of wild type Tub4. Spc97-5FLAG efficiently immunoprecipitated the mutant Tub4 proteins together with WT Tub4 (Tub4-6HA) and Spc98 (Figure 6Aii). Similarly, Tub4 variants and Spc97-5FLAG were co-immunoprecipitated by Tub4-6HA (Figure 6Aiii). These data suggest efficient incorporation of wild type Tub4 and the phospho-mimicking mutant Tub4 proteins into common complexes.

Next, we used the *TUB4-AID SPC97-3HA* strain to investigate stability of mutant Tub4 proteins and complex formation in the absence of WT Tub4. After degradation of Tub4-AID (Figure 6B, top), cells were lysed with glass beads and the level of Tub4, Spc97, Spc98 and Spc110 was determined by immunoblotting (Figure 6Ai). As a control for the efficiency of the extraction of the glass bead method, yeast cells were treated with TCA and proteins were solubilised in SDS-urea buffer (Figure S7A, B) [51]. The TCA-based extraction method was developed to solubilise integral membrane proteins and thus extracts proteins efficiently from yeast cells [5]. Both protocols gave similar results, indicating that the glass bead method extracted Tub4 complex proteins efficiently from cells. The levels of the three mutant Tub4 proteins were in both extractions reduced to 60–30% of wild type Tub4 (Figure 6B, C and Figure S7). In addition, Spc98 levels were reduced in “null”, *tub4-S100E* and *tub4-S360D* cells (Figure 6C; Figure S7B), suggesting that Tub4 stabilizes Spc98. Taken together, we suggest that the mutant Tub4 proteins are unstable but become stabilized by the coexpression of WT *TUB4* (Figure 6A), possibly because of the formation of complexes containing both, WT and mutant Tub4 proteins.

Using anti-HA antibodies, we immunoprecipitated Spc97-3HA from the extracts of *TUB4-AID tub4 SPC97-3HA* cells (Figure 6B, D) to investigate the complex formation in the absence of WT Tub4 (Tub4-AID). We also tested the co-immunoprecipitation of the Tub4 receptor Spc110 to see whether this interaction is regulated by Tub4 phosphorylation [8], [15]. After depletion of Tub4-AID (Figure 6B, top), with anti-HA antibodies we immunoprecipitated similar levels of Spc97-3HA from all *TUB4-AID SPC97-3HA* cell extracts (Figure 6B, D). All three phospho-mimicking mutant Tub4 proteins could be co-immunoprecipitated with Spc97-3HA, together with Spc98 and Spc110 (Figure 6B, D). However, the amount of the co-immunoprecipited mutant Tub4 proteins was lower than in wild-type Tub4 protein, indicating that there was less Tub4 complexes formed. This may reflect the observation that in all three phospho-mimicking *tub4* mutant cells the overall cellular Tub4 protein levels were lower than in wild-type *TUB4* cells.

Variant levels of co-immunoprecipitated Spc110 were also observed in the three phospho-mimicking *tub4* mutant cells. Tub4-S100E had no or only a moderate influence on the co-immunoprecipitation between Spc97-3HA and Spc110. Spc110 was slightly better co-immunoprecipitated by Spc97-3HA from extracts of *tub4-S74E* cells than from *TUB4* wild type extracts (Figure 6D), despite reduced cellular Spc110 protein level and slightly reduced Tub4-S74E complex formation. Tub4-S74 phosphorylation may therefore increase the affinity of the Tub4 complex for Spc110. In contrast, in *tub4-S360D* cells, far less Spc110 and Spc98 proteins were co-immunoprecipitated with Spc97-3HA (Figure 6D), suggesting that the lower amounts of Tub4 complex in *tub4-S360D* cells limits interaction with Spc110.

Taken together, we conclude that in the absence of wild-type Tub4 protein, phospho-mimicking Tub4 mutant proteins are still able to form complexes with Spc97 and Spc98, although the efficiency was reduced in comparison to wild type Tub4 (see Discussion).

### Overexpression of *tub4-S360D* restores viability

**Figure 7:**
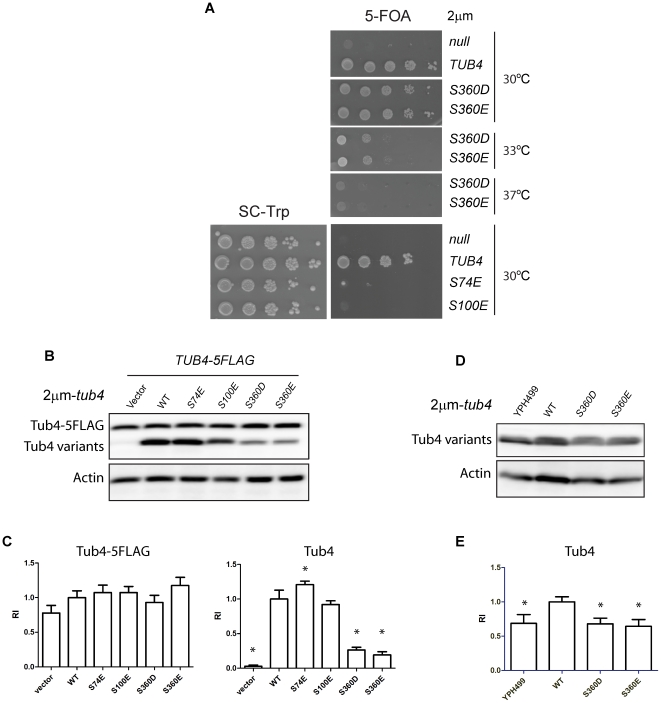
Overexpression of tub4-S360D restores viability. (A) High gene copy of *tub4-S360D* restores growth of cells. *tub4Δ* pRS424-*tub4-S360D* or *tub4Δ tub4-S360E* cells grew at 23°C and 30°C but show reduced or no growth at 33°C or 37°C. In contrast, *tub4Δ* pRS424-*tub4-S74E* and *tub4Δ* pRS424-*tub4-S100E* cells were not viable at 23°C (not shown) or 30°C. (B) Immunoblot blot analysis of yeast cell extracts from *TUB4-5FLAG* cells carrying the indicated mutant *tub4* alleles on the 2 µm plasmid pRS424. The upper blot was developed with anti-Tub4 antibodies. The anti-actin blot was used as loading control. (C) Quantification of the signals of (B). The graph shows the quantification of anti-Tub4-FLAG and anti-Tub4 signals from three independent experiments, normalized to actin levels. Shown is the mean of Tub4 levels with standard deviation. Significance of the difference between wild type and mutants at *p*<0.05 was determined by one-way ANOVA and is indicated by an asterisk. (D) Immunoblot blot analysis of yeast cell extracts from YPH499 *TUB4* wild type cells, and *tub4Δ* cells with 2 µm-*TUB4* (“WT”), 2 µm-*tub4-S360D* and 2 µm-*tub4-S360E*. The anti-actin blot was used as loading control. (E) Quantification of the signals of (D). The graph shows the quantification of anti-Tub4 signals from three independent experiments, normalized to actin levels. Shown is the mean of Tub4 levels with standard deviation. Significance of the difference between YPH499 and 2 µm strains at *p*<0.05 was determined by one-way ANOVA and is indicated by an asterisk.

We asked whether overexpression of *TUB4* mutants will restore viability. This is expected if the reason for the lethal phenotype is mainly the reduced stability of the mutant protein. Interestingly, overexpression of *tub4-360E* or *tub4-S360D* from a high copy 2 µm plasmid restored growth of *tub4Δ* cells at 23°C and 30°C but not at higher temperatures (Figure 7A). In contrast, *tub4Δ* cells with 2 µm-*tub4-S74E* or 2 µm-*tub4-S100E* failed to grow despite high protein levels of the respective Tub4 mutant proteins (Figure 7A–C).

Immunoblotting of Tub4 in *tub4Δ* 2 µm-*tub4-S360E* and 2 µm-*tub4-S360D* cells indicated that Tub4-S360D and Tub4-S360E can accumulate up to levels comparable to non-overexpressed wild type Tub4 (YPH499) but moderately lower than overexpressed wild-type Tub4 (2 µm-*TUB4*) (Figure 7D, E). Interestingly, in the presence of Tub4 (*TUB4-5FLAG*), the level of Tub4-S360E/D mutant protein (2 µm-*tub4-S360E* and 2 µm-*tub4-S360D*) was lower than WT Tub4 expressed from 2 µm-*TUB4* plasmid (Figure 7B, C). This is in contrast to non-overexpressed *tub4-S360D* in the presence of *TUB4* that stabilizes Tub4-S360D (Figure 6A). We propose that the excess of the overproduced Tub4-S360D/E that is not incorporated into the Tub4 complex is degraded, while the fraction in the Tub4 complex is stabilized. In summary, the lethal phenotype of *tub4-S360D/E* cells can be partially explained by the reduced Tub4 protein levels. In contrast, impaired viability of *tub4-S74E* and *tub4-S100E* cells is probably cause by defects in Tub4 function.

### Binding of Spc97, Spc110 and mutated Tub4 proteins to SPBs in *tub4-S74E, tub4-S100E* and *tub4-S360D* cells

**Figure 8:**
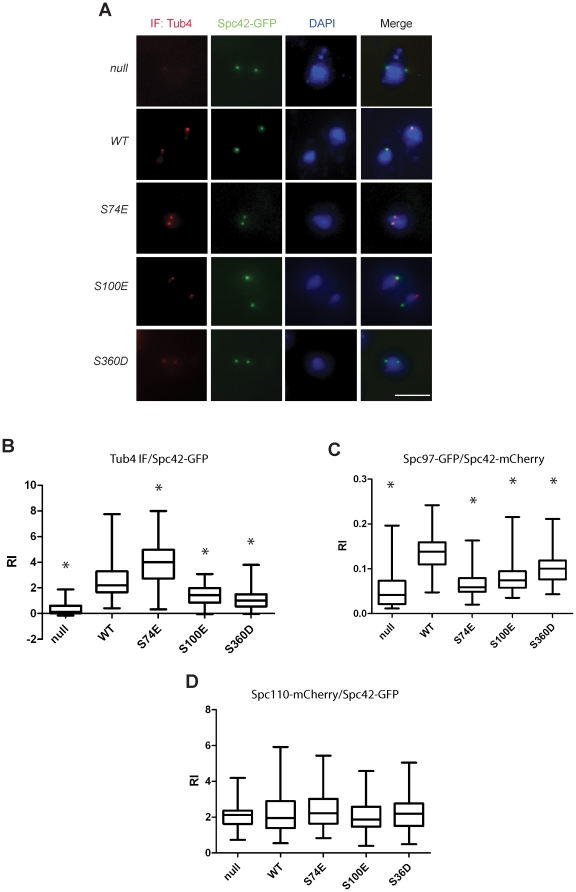
SPB localization of Tub4-S74E, Tub4-S100E and Tub4-S360D. (A) *TUB4-AID SPC42-GFP* cells with the empty plasmid pRS305 (null), or *TUB4* (WT), *tub4-S74E*, *tub4-S100E* or *tub4-S360D* on the integration plasmid pRS305 were incubated with IAA to induce degradation of Tub4-AID. Cells were fixed and then analyzed by indirect immunofluorescence for Tub4 at SPBs. DNA was stained with DAPI. Scale bar: 5 µm. (B) Quantification of the Tub4 SPB signal of cells from (A). Signal intensities were background-subtracted and normalized against the Spc42-GFP signal. Significance of the difference between wild-type and mutant Tub4 signals (p<0.001) was determined by one-way ANOVA and is indicated by an asterisk. (C and D) *TUB4-AID SPC42-mCherry SPC97-GFP* cells and *TUB4-AID SPC42-GFP SPC110-mCherry* cells with the empty plasmid pRS305 (“null”), or *TUB4* (WT), *tub4-S74E*, *tub4-S100E* and *tub4-S360D* on the integration plasmid pRS305 were incubated with IAA to induce degradation of Tub4-AID. Spc97-GFP and Spc110-mCherry signals at SPBs were determined with fluorescence microscopy and then quantified. (S) Significance of the difference between wild-type and mutants at p<0.05 was determined by one-way ANOVA and is indicated by an asterisk. RI: relative intensity.

Phosphorylation of Tub4 may regulate binding of the Tub4 complex to SPBs, as indicated by the increased interaction between Tub4-S74E and Spc110 (Figure 7). For this reason, we measured the accumulation of Tub4 protein at the SPB. Construction of GFP fusions of the *tub4* phospho-mimicking alleles was not possible due to lethality of the gene fusions. We therefore used indirect immunofluorescence with anti-Tub4 antibodies (Figure 8A). Quantification of the signal showed that Tub4-S74E accumulated more strongly at SPBs than WT Tub4 (Figure 8B). This is consistent with the more efficient co-immunoprecipitation of Spc110 by Spc97 in *tub4-S74E* cells (Figure 6D). However, in comparison to WT Tub4, SPB binding of Tub4-S100E and Tub4-S360D was reduced.

Because conformational changes may affect the accessibility of the anti-Tub4 antibodies to the antigen, we analyzed Spc97-GFP at SPBs. Tub4 and Spc97 only bind to SPBs when they are assembled into the Tub4 complex [5]. Therefore, both the SPB signals for Tub4 and Spc97 should be proportional. A reduction of the Spc97-GFP SPB signal was detected in “null”, *tub4-S74E*, *tub4-S100E* and *tub4-S360D* cells (Figure 8C). For *tub4-S100E* and *tub4-S360D* cells this reduction is consistent with the reduced binding of Tub4 (Figure 8A, B). The distinct behaviour of Tub4-S74E and Spc97-GFP in *tub4-S74E* cells is presently not understood (see Discussion). In summary, the phospho-mimicking mutations (*tub4-S100E* and *tub4-S360D*) reduce the Tub4 and Spc97 signal at SPBs.

The Spc110-mCherry signals at the SPBs in *tub4-S74E*, *tub4-S100E* and *tub4-S360D* cells were similar to *TUB4* in WT cells (Figure 8D), suggesting that phosphorylation of Tub4 has no impact on Spc110 incorporation into SPBs.

## Discussion

In this study we identified more than 50 phosphorylation sites in the Tub4 complex subunits Spc97, Spc98 and Tub4 and the receptors Spc72 and Spc110 (Table S1). Less than one third of these phosphorylation sites have been reported before (Table S1) [24], [25], [36], [37], [52] and only the functional relevance of the phosphorylation sites in Spc110 and the Y445 site in Tub4 have been studied [24], [25]. Thus, our analysis has greatly expanded the regulatory potential of microtubule organization in budding yeast.

Phosphorylation of Tub4 complex components was determined after co-overexpression of *SPC97-TAP*, *SPC98* and *TUB4* [29]. The receptors Spc72 and Spc110 co-purified with Spc97-TAP, indicating that at least a fraction of the Tub4 complex was in a functional state. Because overexpression of *SPC97* and *SPC98* induces microtubule defects [5], [29], some phosphorylations may be the result of checkpoint or stress pathway activation. Checkpoint or stress induced phosphorylations of the Tub4 complex components may ensure microtubule attachment to the SPB, fine-tune microtubule dynamics or induce nucleation of microtubules. These measures would increase the chance of survival of cells with a damaged microtubule cytoskeleton.

Most phosphorylation sites in Spc97 and Spc98 reside in regions outside the highly conserved portions (Figure 1C). This is consistent with a recent report demonstrating that most phosphorylation sites are not conserved in evolution but instead cluster in rapidly evolving disordered regions [37]. None of the identified phosphorylation sites in Spc97 and Spc98 were essential for cell viability. Phosphorylation may fine-tune the function of Spc97 and Spc98 or only the combination of phosphorylation events may be essential for the function of the Tub4 complex.

Three of the phosphorylation sites in Tub4 (S74, S100 and S360) turned out to be essential for viability of cells. Of these, only Tub4-S360 is highly conserved between γ-tubulins from yeast to mammals (Figure 1E). Tub4-S360 matches the minimal consensus motif of Cdk1 (S/T-P) and was identified as an in vivo Cdk1 phosphorylation site [37]. Sequence analysis indicates that Tub4-S74 corresponds to the Cdc5 polo-like kinase consensus sequence [30]. In this respect it is interesting that Cdc5 phosphorylates Tub4 *in vitro* (T.L., unpublished). Presently it is unclear which kinase phosphorylates Tub4-S100.

*tub4-S74E*, *tub4-S100E* and *tub4-S360D* cells were able to assemble short bipolar spindles but then arrested in the cell cycle in metaphase due to SAC activation. EM analysis of *tub4* phospho-mimicking mutant cells revealed defects in nuclear microtubules organization rather than a microtubule nucleation defect as this was seen in *TUB4-AID* depleted cells (Figures 4 and 5). The phenotypes of *tub4-S74E*, *tub4-S100E* and *tub4-S360D* cells were distinct from *tub4-34* cells that fail to organize the full set of microtubules at the newly assembled SPB [17]. However, the nuclear microtubule defects in *tub4-S74E* cells were similar to the defects observed in conditional lethal *spc97-14* cells [6], raising the possibility of similar functional consequences of these mutations for Tub4 complex function.

Assays regarding protein levels, Tub4 complex assembly and SPB binding gave first insights into the defects of the phospho-mimicking Tub4 mutant proteins. Tub4 levels and Tub4 complex formation were strongly reduced in *tub4-S360D* cells (Figure 6). One possibility is that Tub4 complex formation is impaired in *tub4-S360D* cells triggering degradation of free Tub4. In this model free Tub4 and free Tub4-S360D have similar half life. Alternatively, or in addition, free Tub4-S360D is much less stable than free wild type Tub4, thus shifting in *tub4-S360D* cells the equilibrium towards Tub4 complex disassembly. Expression of WT *TUB4* in cells with single copy *tub4-S360D* restored the levels of the mutant Tub4 protein (Figure 6A). In these cells the mutant Tub4-S360D was efficiently incorporated into Tub4 complexes. In contrast, Tub4-S360D (2 µm-*tub4-S360D*) overproduced in a WT *TUB4* background accumulated much lower than overproduced WT *TUB4* (Figure 7B, 2 µm-*TUB4*). These observations are consistent with the notion that free Tub4-S360D is less stable than free WT Tub4, while Tub4-S360D in the Tub4 complex is stabilized. We suggest that excess Tub4, which accumulates in the G1 phase due to cell cycle specific transcription of the *TUB4* gene [17], [53], is to some extent degraded in response to increasing Cdk1 phosphorylation. Of note here is that microtubule nucleation in budding yeast is probably restricted to G1 phase when the SPB duplicates [17]. Thus, it may be beneficial for cells to remove excess Tub4 from the other phases of the cell cycle.

High gene dosage of *tub4-S360E/D* rescued the lethality of *tub4Δ* cells at 23°C. Therefore, Tub4-S360E/D is able to fulfil the essential functions of Tub4 when protein levels are restored to WT Tub4 levels. However, 2 µm-*tub4-S360E/D tub4Δ* cells were inviable at 37°C. This either means that an essential function is compromised in Tub4-S360E/D at higher temperatures or that degradation of Tub4-S360E/D is further increased at 37°C.

As a consequence of diminished Tub4-S360D levels, Spc98 was also reduced. Such a reduction of Spc98 was also observed in *TUB4-AID* depleted “null” cells, suggesting a role of Tub4 in Spc98 stabilization. Coupling Spc98 stability to Tub4 levels helps to counteract accumulation of toxic Spc98 [5]. In fact, co-overexpression of *SPC98* together with *TUB4* rescues cells from the lethal effects of *SPC98* overexpression [5].

Tub4-S74E accumulated to about 60% of the cellular WT Tub4. Despite this 40% reduction, Tub4-S74E assembled together with Spc97-3HA efficiently into complexes (∼75% of WT Tub4). The 3-fold excess of Tub4 over Spc97 [54] probably explains why moderately reduced Tub4 levels did not strongly affect Tub4 complex formation, assuming that the affinity of the mutant Tub4 protein for Spc97 and Spc98 was not altered. Importantly, 2 µm-*tub4-S74E*, which increases Tub4-S74E levels above WT Tub4, did not allow growth of *tub4Δ* cells. These data argue that a failure of an essential Tub4 function and not the lower amount of cellular Tub4 causes the defect in *tub4-S74E* cells. Tub4-S74E complexes were found to interact better with Spc110 in co-immunoprecipitation experiments than WT Tub4 complex. In addition, a higher Tub4-S74E signal was determined at SPBs than for Tub4. Thus, the binding affinity of the Tub4 complex to the nuclear receptor Spc110 may be increased in *tub4-S74E* cells. Puzzlingly however, Spc97-GFP binding to SPBs was reduced in *tub4-S74E* cells. It is possible that the GFP tag hinders the interaction between Spc97 and Tub4-S74. Alternatively, Tub4-S74E at SPBs may be more accessible for the polyclonal Tub4 antibodies than WT Tub4. In the latter case, we have to assume that the conformational change of Tub4-S74E or the Tub4-S74E complex is sufficiently strong to allow the anti-Tub4 antibodies a much better access to SPBs than in wild type cells.

Tub4-S100E assembled together with Spc97 and Spc98 with slightly reduced efficiency into complexes compared with WT Tub4. When analyzed by indirect immunofluorescence, clearly less Tub4-S100E signal was detected at SPBs than WT Tub4. Consistent with this result, the SPB signal of Spc97-GFP was reduced in *tub4-S100E* cells (Figure 8). Thus, Tub4-S100 phosphorylation may regulate binding of the Tub4 complex to SPBs. In this respect it is interesting that *tub4-S100E* cells assembled a bipolar spindle later in the cell cycle than wild type *TUB4* cells or the *tub4-S74E* and *tub4-S360D* mutant cells. Diminished Tub4 nucleation activity in early phases of the cell cycle would explain this phenotype of *tub4-S100E* cells. Phosphorylation of Tub4-S100 may delay microtubule nucleation in G1 and early S phase. This would prevent the kinesin-5 motor proteins Cin8 and Kip1 from assembling a bipolar spindle before end of S phase [55].

Our analysis has shown that phosphorylation of Tub4 at different sites has the potential to regulate microtubule organization in the model organism budding yeast. It will be interesting to see whether this is also the case in higher eukaryotes and whether the conserved S360 residue in human γ-tubulin contributes to the phospho-regulation of human γ-tubulin complexes.

## Materials and Methods

### Strain constructions

Strains and plasmids of this study are listed in Table S2 and S3. All strains are derivatives of S288c. Gene deletion, epitope tagging of genes at their endogenous loci and mutagenesis were performed using standard techniques [51], [56]. The red fluorescent mCherry and eqFP611 were used to mark SPBs through a fusion with *SPC42* [26]. *GFP-TUB1* strains were constructed using integration plasmids [48].

To construct an auxin-inducible degron-Tub4 (*TUB4-AID*) strain, cells of YPH499 [57] were transformed with an integration plasmid encoding the F-box transport inhibitor response 1 (TIR1) gene driven by the *ADH1* promoter. Endogenous *TUB4* was tagged at its 3' end with the IAA17 auxin-binding element (AID) of *Arabidopsis thaliana* [47].

*TUB4*, *SPC97* and *SPC98* were cloned into the integration vector pRS305, the centromere-based plasmid pRS316 or the 2 µm plasmid pRS424 [57], [58]. Mutations in genes were introduced by PCR-directed mutagenesis and confirmed by DNA sequencing. Selected phosphorylation sites of the mass spectrometry analysis were mutated to alanine (A) to avoid phosphorylation or mutated to aspartic acid (D) or glutamatic acid (E) to mimic phosphorylation. Transformants were tested for growth on 5-fluoroorotic acid (5-FOA) plates that select against *URA3*-based plasmids.

For immunoprecipitation experiments integration plasmids encoding the Tub4 variants were integrated into strain YPH499 in which the endogenous *TUB4* was fused in frame at its 3' end with six copies of the hemagglutinin epitope HA (*TUB4-6HA*) or *SPC97* with five copies of the FLAG epitope (*SPC97-5FLAG*).

### Growth conditions of yeast cells

For the identification of phosphorylation sites of the Tub4 complex, we used strain YDH002-1 that allowed galactose-induced over-expression of Tub4 complex components and subsequent purification of Spc97-TAP associated proteins. Cells were grown in yeast peptone dextrose (YPD) medium to an OD_600_ of 2–3 and shifted to medium with 2% galactose as sole carbon source for 3-6 h. Thirty minutes before harvesting, phosphatase inhibitor cocktail (2 mM sodium orthovanadate, 5 mM sodium fluoride, 2 mM sodium pyrophosphate, 2 mM glycerol–2-phosphate) was added to the medium. Cell pellets were frozen in liquid nitrogen and stored at −20°C.

For fluorescence and immunofluorescence microscopy (Figures 4B and 8A), *TUB4-AID* cells (5×10^6^ cells/ml) were pre-grown at 30°C in filter-sterilized YPD with additional 0.1 mg/l adenine (YPAD). Cells were arrested in G1 by treatment with 10 µg/ml α-factor (Sigma-Aldrich) for 2–2.5 h at 30°C until >95% of cells showed a mating projection. Thirty minutes before release of the G1 arrest, 0.5 mM IAA was added to deplete Tub4-AID. Cells were then released from G1 arrest with pre-warmed YPAD medium containing IAA. To activate spindle assembly checkpoint (Figure S4), 1.5 µg/ml nocodazole was added together with IAA when cells were released from G1 arrest.

For the measurement of spindle length of cells in metaphase, Gal1-*CDC20 GFP-TUB1* cells pre-grown in yeast peptone raffinose galactose (YPRG) medium were synchronized with α-factor (10 µg/ml) for 2–2.5 h at 30°C until >95% of cells showed a mating projection. After release of cells from α-factor block with YPAD medium, cells were arrested in metaphase due to repression of Gal1-*CDC20* expression [59].

For immunoprecipitation of proteins from Tub4-AID cells (Figure 6B), Asynchronous cells were grown in YPAD at 30°C to mid log-phase. Depletion of Tub4-AID was achieved by incubation of cells with 0.5 mM IAA for 2 h.

### Purification of Tub4 complex/Spc97-TAP

30 g frozen cell pellet were resuspended in lysis buffer (20 mM Tris/HCl pH 7.5, 5% glycerol, 10 mM EDTA, 0.1 mM EGTA, 0.1 M NaCl, 0.1% NP-40) supplemented with 2x phosphatase inhibitor cocktail and protease inhibitors (Roche) and lysed using glass beads. The lysate was clarified by centrifugation and the supernatant applied to an IgG-Sepharose column (1 ml IgG-Sepharose 6 Fast Flow, GE Healthcare). The column was washed with a minimum of 10 column volumes each of the following buffers: wash buffer 1 (50 mM HEPES/KOH pH7.9, 0.6 M potassium acetate, 0.5 mM EDTA, 10 mM β-mercaptoethanol, 0.1% NP-40), wash buffer 2 (TBS buffer plus 0.1% Tween), wash buffer 3 (5 mM ammonium acetate, pH5.5). Proteins were eluted with 0.2 M acetic acid pH3.4 and immediately neutralized with approx. 0.3 volumes 1 M Tris base, stabilized with 1x phosphatase inhibitor cocktail and lyophilized.

### In-solution digestion and phosphopeptide enrichment

The purified sample was digested with Lys-C and trypsin. The lyophilized sample was dissolved in 6 M urea/2 M thiourea, reduced by 1 mM dithiotreitol for 30 min, alkylated with 5 mM iodoacetamide for 45 min and digested with endoproteinase Lys-C (WACO) for 3 h at room temperature. After 4-fold dilution with 20 mM ammonium bicarbonate, the solution was further incubated with trypsin (Promega) for 16 h at room temperature. The protease to protein ratio was 1 to 100 in both cases. Phosphopeptides were enriched by Titansphere-chromatography (TiO_2_) as described [60] with minor modifications. Acidified peptide fractions were incubated with approximately 5 µl of a 1∶1 slurry of Titansphere-material (10 µm, GL Sciences, Japan) and 0.5 g/l 2,5-dihydrobenzoic acid (DHB) in 80% acetonitrile (ACN)/0.1% trifluoroacetic acid (TFA) under rotation for 30 min. Beads were washed once with 100 µl 40% ACN/0.1% TFA and twice with 80% ACN/0.1% TFA and then transferred to home-made C8-Stage Tips in 200 µl pipet-tips. The collected beads in the C8 tip were washed once more with 80% ACN/0.1% TFA. Phosphopeptides were eluted directly into a 96-well plate with 2×20 µl of 25% ACN in 5% ammonia-water solution (pH∼11) and dried in a speed-vac.

### Nano LC-MS/MS analysis

The dried yeast phosphopeptide mixtures were acidified with 5% ACN/0.3% TFA to an end volume of 10 µl, transferred to a 96-well plate and analyzed by online nanoflow liquid chromatography tandem mass spectrometry (LC-MS/MS) as described previously [60] with a few modifications. In brief, all nano LC-MS/MS-experiments were performed on an EASY-nLC™ system (Proxeon Biosystems, Odense, Denmark) connected to the LTQ-Orbitrap XL (Thermo Electron, Bremen, Germany) through a nanoelectrospray ion source.

5 µl of each phosphopeptide fraction was auto-sampled and directly separated in a 15 cm analytical column (75 µm inner diameter) in-house packed with 3-µm C18 beads (Reprosil-AQ Pur, Dr. Maisch) with a 90 min gradient from 5% to 30% ACN in 0.5% acetic acid at a flow rate of 250 nl/min. The effluent from the HPLC was directly electrosprayed into the mass spectrometer by applying 2.2 kV to a platinum-based liquid-junction.

The LTQ-Orbitrap XL instrument was operated in the data-dependent mode to automatically switch between full scan MS and MS/MS acquisition. Survey full scan MS spectra (from m/z 300 – 2000) were analyzed in the orbitrap detector with resolution R = 60,000 at m/z 400 (after accumulation to a ‘target value’ of 1e6 in the linear ion trap). The ten most intense peptide ions with charge states ≥2 were sequentially isolated to a target value of 5e3 and fragmented by low-energy collision-induced dissociation (CID) with a normalized collision energy setting of 35%. Multi-stage activation (MSA or pseudo-MS3) was enabled for the neutral loss of 32.66 Da, 48.99 Da and 97.97 Da. The resulting fragment ions were detected by the electron multipliers of the LTQ. The ion selection threshold was 500 counts and the maximum allowed ion accumulation times were 500 ms for full scans and 250 ms for HCD.

Standard mass spectrometric conditions for all experiments were: spray voltage, 2.2 kV; no sheath and auxiliary gas flow; heated capillary temperature, 200^o^C. For all full scan measurements with the Orbitrap detector a lock-mass ion from ambient air (m/z 445.120024) was used as an internal calibrant as described [61].

### Data processing and analysis

Raw Orbitrap full-scan MS and ion trap multi-stage activation MS/MS spectra were processed by MaxQuant as described [62], [63]. Briefly, all identified isotope clusters were quantified, accurate precursor masses determined based on intensity-weighing precursor masses over the entire LC elution profiles and MS/MS spectra were merged into peak-list files. Peptides were identified from MS/MS spectra by searching against a concatenated forward and reversed version (the target-decoy database, [64]) of the yeast ORF database (*Saccharomyces cerevisiae* Genome Database SGDTM at Stanford University; http://www.yeastgenome.org) using the Mascot (Matrix Science) search engine. Protein sequences of common contaminants, for example, human keratins and porcine trypsin were added to the database. The initial mass tolerance in MS mode was set to 7 p.p.m. and MS/MS mass tolerance was 0.5 Da. We required strict trypsin enzyme specificity and allowed for up to two missed cleavage sites. Cysteine carbamidomethylation (Cys +57.021464 Da) was searched as a fixed modification, whereas N-acetylation of proteins (N-term +42.010565 Da), N-pyroglutamine (-17.026549 Da), oxidized methionine (+15.994915 Da) and phosphorylation of serine, threonine and tyrosine (Ser/Thr/Tyr +79.966331 Da) were searched as variable modifications.

The resulting Mascot result files were loaded into the MaxQuant for further processing. In MaxQuant we fixed the estimated false discovery rate (FDR) of all peptide and protein identifications at 1%, by automatically filtering on peptide length, mass error precision estimates and Mascot score of all forward and reversed peptide identifications. Finally, to pinpoint the actual phosphorylated amino acid residue(s) within all identified phospho-peptide sequences in an unbiased manner, MaxQuant calculated the localization probabilities of all putative serine, threonine and tyrosine phosphorylation sites using the PTM score algorithm as described [60].

### Growth assay

Yeast cells in the early log phase were adjusted to an OD_600_ of 1 with PBS. Ten-fold serial dilutions of cells were spotted onto the indicated plates. Plates were incubated as indicated in the figure legends.

### Protein sequence alignment

Protein sequences of Tub4, Spc97 and Spc98 and their homologues in selected organisms were aligned using T-Coffee [65]. The conserved regions were highlighted with CINEMA in Utopia Protein Analysis Suite [66]. Domain positions were illustrated according to **γ**-TuSC binding studies of Spc72- and Spc110-truncated forms and to the segmentation of Spc97 and Spc98 [15], [40], [67], [68].

### Antibodies

Affinity-purified anti-Tub4, anti-Spc98 and anti-Spc110 antibodies were described previously [5], [16], [69]. Mouse monoclonal anti-HA antibody 12CA5 and anti-FLAG was used to detect and immunoprecipitate Spc97-3HA/Tub4-6HA and Spc97-5FLAG, respectively. Secondary antibodies used were HRP-conjugated goat anti–mouse, goat anti–rabbit, and rabbit anti–goat IgGs (Jackson ImmunoResearch Laboratories, Inc.).

### TCA extraction of yeast cells

To prepare whole cell lysates, 2-3 OD_600_ of late-log phase liquid culture were resuspended in 0.2 M NaOH and incubated on ice for 10 min. 150 µl 55% (w/v) TCA was added, the solutions were mixed and incubated for 10 min on ice. After centrifugation the supernatant was removed. The protein pellet was resuspended in High Urea (HU) buffer (8 M urea, 5% SDS, 200 mM Tris-HCl pH 6.8, 0.1 mM EDTA, 100 mM DTT, bromophenol blue) and heated at 65°C for 10 min.

### Immunoprecipitation expeeriments

Cell pellets were resuspended in 1 ml lysis buffer (50 mM Tris-HCl pH7.5, 150 mM NaCl, 1 mM EDTA, 10% glycerol, 1 mM DTT, complete EDTA-free protease inhibitor cocktail (Roche), 1 mM PMSF) and were lysed with acid-washed glass beads (Sigma-Aldrich) in a FastPrep FP120 Cell Disrupter (Thermo Scientific). Cell lysates were incubated with 0.1% Triton X-100 for 15 min and then clarified by centrifugation at 10,000 g for 10 min. The cleared lysates were incubated at 4°C with anti-FLAG or anti-HA antibody for 1.5 h and then with Protein G-coated Dynabeads for another 1.5 h. After washing with lysis buffer, proteins were eluted from beads by boiling in Laemmli buffer for 5 min at 95°C.

### Quantification of immunoblots

The signal intensities of protein bands on immunoblots were quantified with ImageJ (NIH). Signal intensities were corrected against the membrane background. The intensities of input proteins and immunoprecipitated proteins were normalized with actin and Spc97-3HA respectively. The value of *TUB4* wild type was set as reference to one. The variance and standard error for each data set (n>3) and significance at p<0.05 were determined by one-way ANOVA.

### Fluorescence microscopy

Cells fixed with 4% paraformaldehyde were incubated with 1 µg/ml 4',6-diamidino-2-phenylindole (DAPI, Sigma-Aldrich) for 1 h at room temperature. Z-stack images with 21 0.3-µm steps (2×2 binning) were acquired at room temperature with a wide-field epifluorescence microscope (Axiophot; Carl Zeiss MicroImaging, Inc.) equipped with a 100x NA 1.45 Plan-Fluar oil immersion objective (Carl Zeiss MicroImaging, Inc.) and a CCD camera (Cascade 1K; Photometrics). The operation was controlled with MetaMorph software (Universal Imaging Corp.). Images were processed with ImageJ (NIH). The phenotypes were determined for 150–250 cells per strain. Maximum projections of stacks are shown (Figure 4B). No manipulations other than contrast and brightness adjustments were used.

Anti-Tub4 immunofluorescence was performed as described [17]. Rabbit anti-Tub4 (gift from J. Kilmartin) and Alexa 488-conjugated anti-rabbit IgGs (Molecular Probes) were used as primary and secondary antibodies, respectively. DNA was stained with DAPI. Z-stack images with 21 0.3 µm steps (2×2 binning) were acquired at room temperature with DeltaVision (Applied Precision) equipped with GFP and TRITC filters (Chroma Technology Corp.), a 100x NA 1.4 plan Apo oil immersion objective (IX70; Olympus), and a CCD camera (CoolSNAP HQ; Roper Scientific). Figure 8A shows maximum projected images. Images were processed and analyzed in ImageJ (NIH). The quantification of background intensity, fluorescence intensity of Spc42-GFP and immuno-stained Tub4 was performed on planes having SPBs in focus. The variance and standard error for each data set (n = 50) and significance at p<0.05 were determined by one-way ANOVA.

### Metaphase spindle length and cytoplasmic microtubule length measurements

Spindle length was determined by measuring the x-y-z coordinates of in-focus SPBs with ImageJ. The distance between SPBs was calculated with Excel (Microsoft) using the equation: [(x_2_−x_1_)^2^+(y_2_−y_1_)^2+^(z_2_−z_1_)^2^]^1/2^ = D. Cytoplasmic microtubule length was determined by measuring the x-y-z coordinates from the beginning (SPB) to the end at the cell cortex. The variance and standard error for each data set (n>100) and significance at p<0.05 were determined by one-way ANOVA.

### Electron microscopy

Yeast cells were high-pressure frozen, freeze-substituted, sectioned and stained for electron microscopy as previously described [70], [71]. In brief, cells were collected from YPAD medium by filtration, and the cell paste was frozen with EM PACT2 high pressure freezer (Leica, Austria). The frozen cells were substituted through a freeze-substitution-solution (0.1% glutaraldehyde, 0.2% uranyl acetate and 1% water in acetone) and stepwise infiltrated with Lowicryl HM20 (Polysciences, Inc., Warrington, PA). For polymerization the samples were exposed to UV light for 48 h at -45°C and were gradually warmed up to 20°C. The polymerized cellblocks were serially sectioned at a thickness of 70 nm with a Reichert Ultracut S Microtome (Leica Instruments, Wien, Austria). After post-staining with 2% uranyl acetate and lead citrate, sections were viewed in a CM120 electron microscope (Philips Electronics N.V., Eindhoven, Netherlands), which was operated at 100 kV. Images were captured with a CCD camera (Keen View, Soft Imaging Systems) and viewed with Digital Micrograph software.

## Supporting Information

Figure S1 **Growth test of phospho-mimicking and non-phosphorylatable** ***spc97*** **,** ***spc98*** **and** ***tub4*** **cells.** (A) Summary of growth test of the indicated *spc97* and *spc98* phospho-mimicking and phospho-inhibiting mutants. The *spc97* and *spc98* alleles on pRS305 were integrated into the genome of *SPC97* and *SPC98* shuffle strains. These cells were then tested for growth on 5-FOA plates. (B) Shown is the growth test of phospho-mimicking and non-phosphorylatable *tub4* alleles in *BUB2 MAD2*, *bub2Δ* and *mad2Δ* backgrounds.
**(**TIF**)**

Figure S2 **Phosphorylation sites of Spc97 and Spc98 are mapped on the single γ-TuSC density map.** The single **γ**-TuSC density map is isolated from the reconstructed filament structure of oligomerized **γ**-TuSC (Electron Microscopy Database accession code 1731). The approximate protein boundaries and the domain organization of Spc97 and Spc98 are defined by Segger plug-in of Chimera, which was described previously. The dash line indicates the interface between Spc97 and Spc98 molecules. The identified phosphorylation sites are categorized according to their positions relative to the domain organization.
(TIF)

Figure S3 ***tub4-S100A*** **mutant cells show disorganized nuclear microtubules in the absence of the spindle assembly checkpoint gene** ***MAD2*** **.** (A) Spindle phenotypes of *tub4-S100A mad2Δ* cells. Wild type, *mad2Δ, tub4-S100A* and *tub4-S100A mad2Δ* cells with *SPC42-eqFP611 GFP-TUB1* were synchronized with α-factor and then shifted to 37°C, the restrictive temperature of *tub4-S100A mad2Δ* cells (t = 0). Cells were sampled 90 min after G1 release and analyzed by fluorescence microscopy. Scale bar: 10 µm. (B) Quantification of phenotypes of cells in (A). N>200 cells per strain were analyzed as indicated in the figure. (C) The population of *tub4-S100A mad2Δ* cells with “MT defects” in (B) was subcategorized into five groups.
(TIF)

Figure S4 **Multi-budded cells emerged when spindle assembly check point gene** ***MAD2*** **was deleted.** (A) Micrographs of *TUB4-AID mad2Δ* cells with empty vector (“null”), *TUB4*, *tub4-S74E, tub4-S100E* or *tub4-S360D* were arrested in G1-phase with α-factor. IAA was added 30 min before G1 release by washing cells with YPAD containing IAA (G1 release t = 0). Cells were fixed with 70% ethanol after 3 hours from releasing. (B) The percentage of multi-budded cells in each *TUB4-AID MAD2* cells (blue bar) and *TUB4-AID mad2Δ* cells (red bar) with/without nocodazole treatment.
(TIF)

Figure S5 **Spindle length of metaphase-arrested Gal1-** ***CDC20*** **cells**. Micrographs of Gal1-*CDC20 SPC42-mCherry GFP-TUB1* cells grown for 90 min in glucose medium to repress expression of Gal1-*CDC20* and to induce arrest of cells in metaphase. Genomic DNA was stained with DAPI. Scale bar: 10 µm.
(TIF)

Figure S6 **EM images of microtubule phenotypes of** ***tub4-S74E*** **,** ***tub4-S100E*** **and** ***tub4-S360D*** **cells.** (A-E) *TUB4-AID* cells with the wild type *TUB4 (“WT”)* (A), empty plasmid (“null”; B), *tub4-S74E* (C), *tub-S100E* (D) or *tub4-S360D* (E) were synchronized with α-factor and treated with IAA as described in Figure 4A. 45 or 75 min after the G1 release cells were prepared for thin serial sectioning and observed under electron microscopy as described in Materials and Methods. (A-D) Shown are three consecutive serial sections. The white arrows indicate the position of defective nuclear microtubules. Abbreviations: CP, cytoplasm; cMT, cytoplasmic microtubules; N, nucleus; NE, nuclear envelope; nMT, nuclear microtubules; SPB, spindle pole body. Scale bar: 200 nm.
(TIF)

Figure S7 **Western blot of TCA cell extracts.** (A) Asynchronous *TUB4-AID* cells were grown in YPAD at 30°C to mid log-phase. Depletion of Tub4-AID was achieved by incubation of cells with 0.5 mM IAA for 2 h. After TCA extraction total cell lysates were analyzed by SDS-PAGE and immunoblotting with anti-Tub4, anti-Spc98, anti-Spc110 and anti-HA (Spc97-3HA blot) antibodies. Anti-actin antibodies were used to normalize loading. (B) Protein levels of cell extracts from (A) were quantified and normalized as described in Figure 6. Significance of the difference between wild-type and mutants at p<0.05 was determined by one-way ANOVA and is indicated by an asterisk.
(TIF)

Table S1 **MS/MS identification of phosphorylation sites of Tub4 complex.** Identified phosphorylation sites subjected to growth test are marked in orange. The two sites (S74 and S100) which are focused in this study are marked in red. The phosporylated residues (S, T, Y) in the sequence are marked in red. §The potentil kinases are predicted according to the following consensus sites: Cdk (S/T-P); Plk (Ψ-E/N/D/Q-X-S/T-Ψ)[30]; Ipl (R/K-X-T/S-I/L/V)[34]; Ck2 (S/T-X-X-E/D/pT/pS)[35]. n.d.: not determined. +The exact phosphorylation site cannot be be deduced from MS/MS spectra. # Conserved residue.
(XLSX)

Table S2 **Plasmids used in this study.**
(XLSX)

Table S3 **Strains used in this study.**
(XLSX)
