# Supplementary figures and images for: Phosphorylation of the Yeast γ-Tubulin Tub4 Regulates Microtubule Function
Source: PLoS One. 2011 May 5;6(5):e19700. doi: 10.1371/journal.pone.0019700 (PMC3088709; doi:10.1371/journal.pone.0019700)

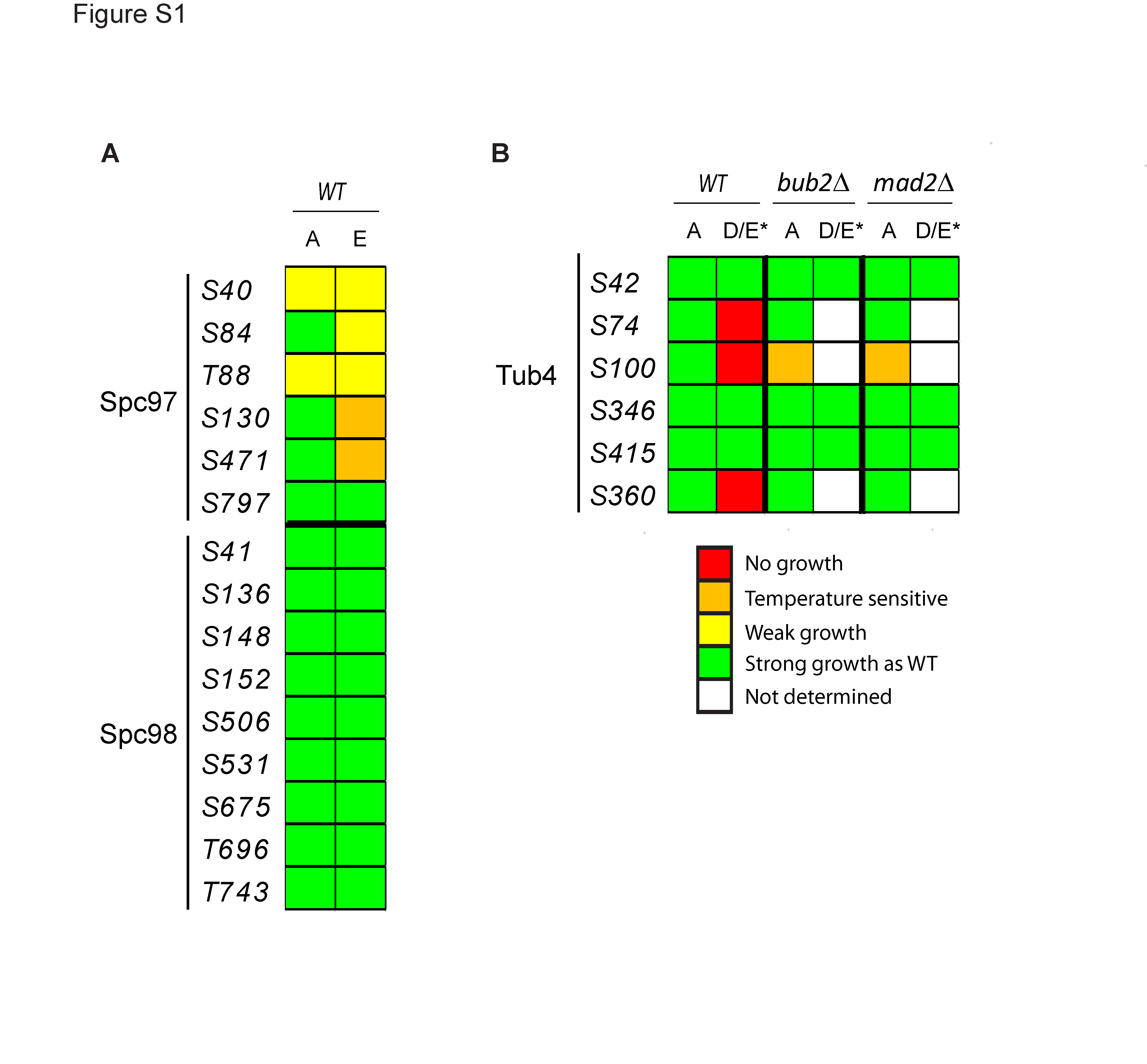

Supplement: Figure S1 — Growth test of phospho-mimicking and non-phosphorylatable spc97 , spc98 and tub4 cells. (A) Summary of growth test of the indicated spc97 and spc98 phospho-mimicking and phospho-inhibiting mutants. The spc97 and spc98 alleles on pRS305 were integrated into the genome of SPC97 and SPC98 shuffle strains. These cells were then tested for growth on 5-FOA plates. (B) Shown is the growth test of phospho-mimicking and non-phosphorylatable tub4 alleles in BUB2 MAD2, bub2Δ and mad2Δ backgrounds. (TIF) [file pone.0019700.s001.tif]

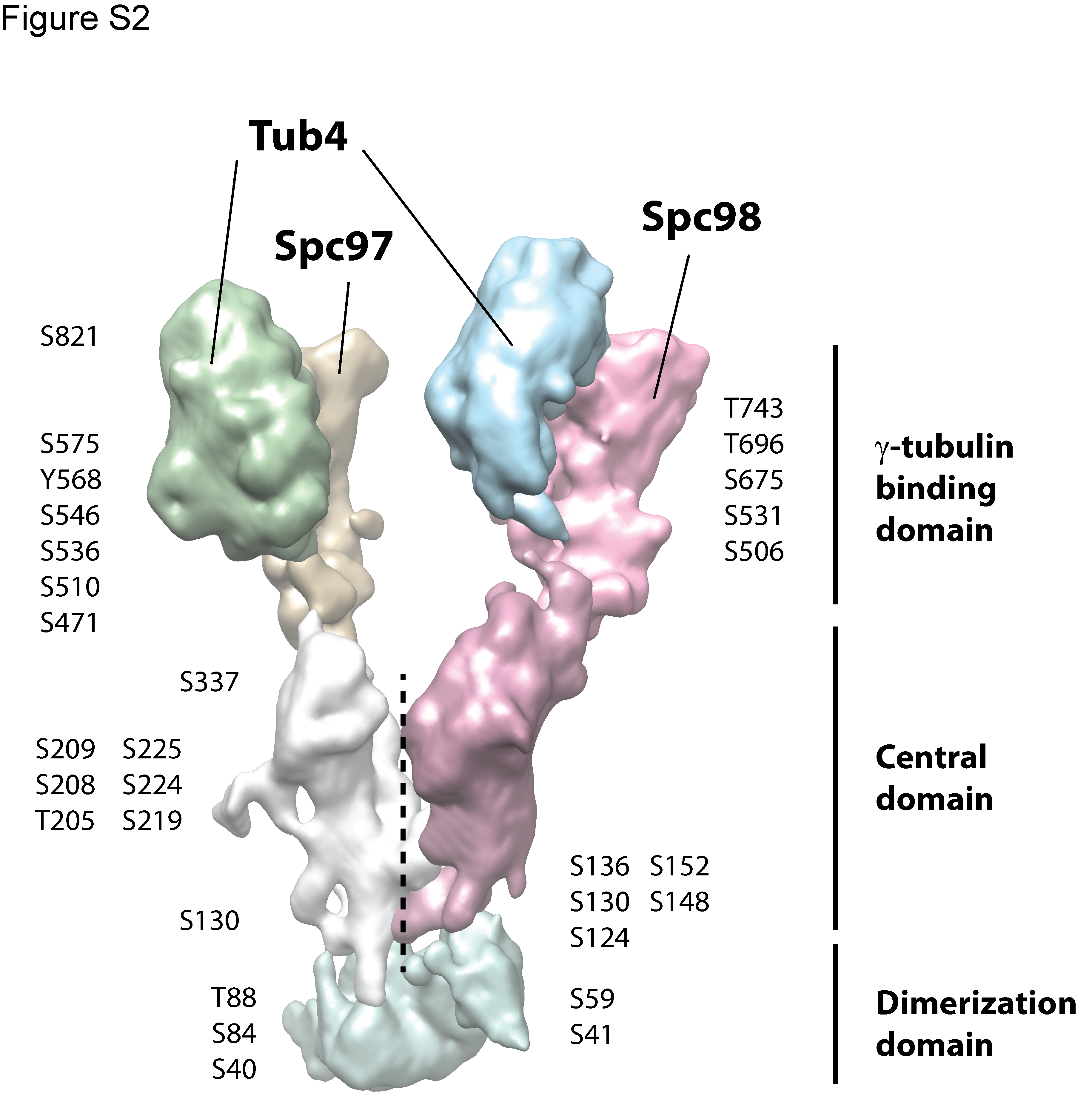

Supplement: Figure S2 — Phosphorylation sites of Spc97 and Spc98 are mapped on the single γ-TuSC density map. The single γ-TuSC density map is isolated from the reconstructed filament structure of oligomerized γ-TuSC (Electron Microscopy Database accession code 1731). The approximate protein boundaries and the domain organization of Spc97 and Spc98 are defined by Segger plug-in of Chimera, which was described previously. The dash line indicates the interface between Spc97 and Spc98 molecules. The identified phosphorylation sites are categorized according to their positions relative to the domain organization. (TIF) [file pone.0019700.s002.tif]

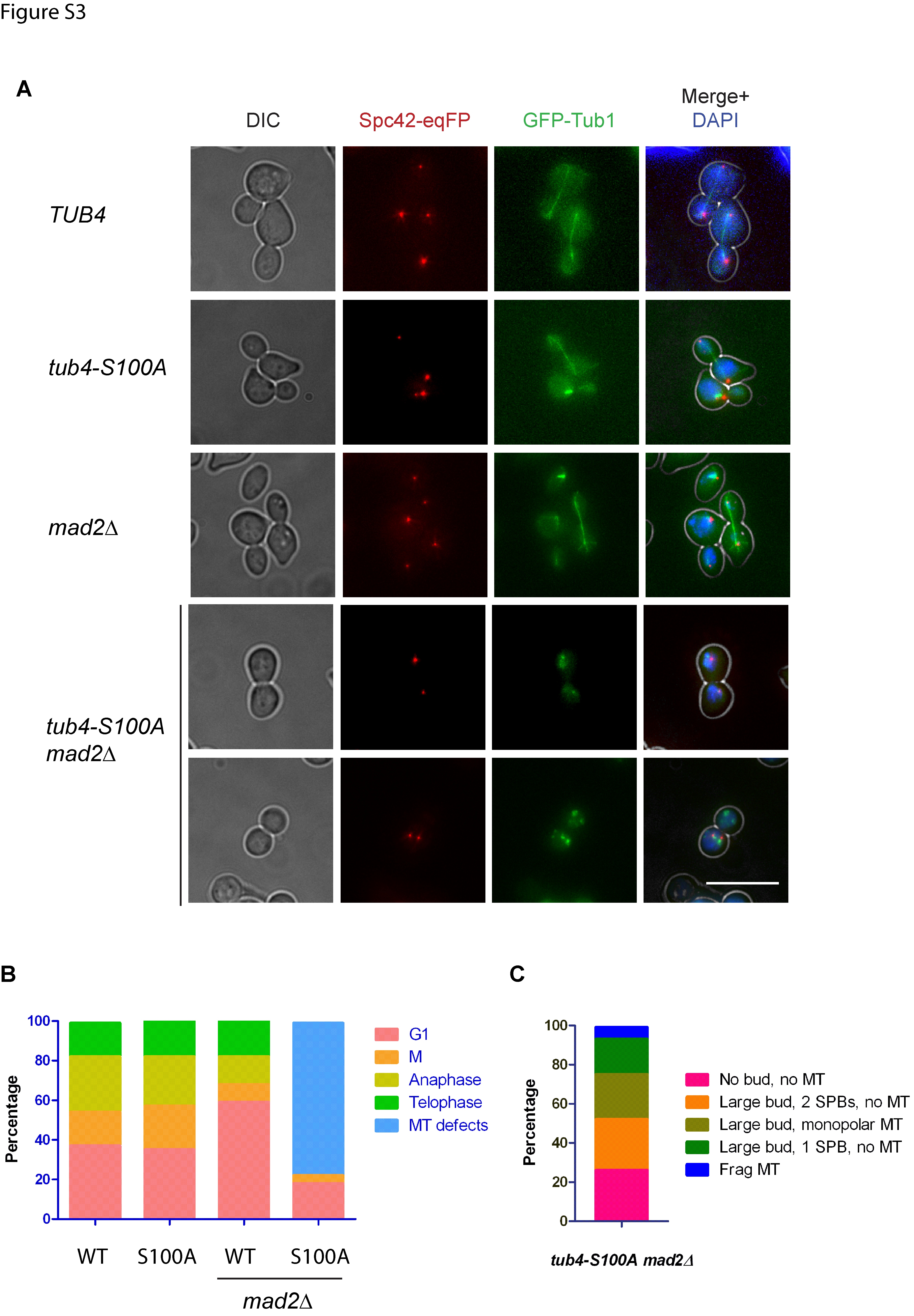

Supplement: Figure S3 — tub4-S100A mutant cells show disorganized nuclear microtubules in the absence of the spindle assembly checkpoint gene MAD2 . (A) Spindle phenotypes of tub4-S100A mad2Δ cells. Wild type, mad2Δ, tub4-S100A and tub4-S100A mad2Δ cells with SPC42-eqFP611 GFP-TUB1 were synchronized with α-factor and then shifted to 37°C, the restrictive temperature of tub4-S100A mad2Δ cells (t = 0). Cells were sampled 90 min after G1 release and analyzed by fluorescence microscopy. Scale bar: 10 µm. (B) Quantification of phenotypes of cells in (A). N>200 cells per strain were analyzed as indicated in the figure. (C) The population of tub4-S100A mad2Δ cells with “MT defects” in (B) was subcategorized into five groups. (TIF) [file pone.0019700.s003.tif]

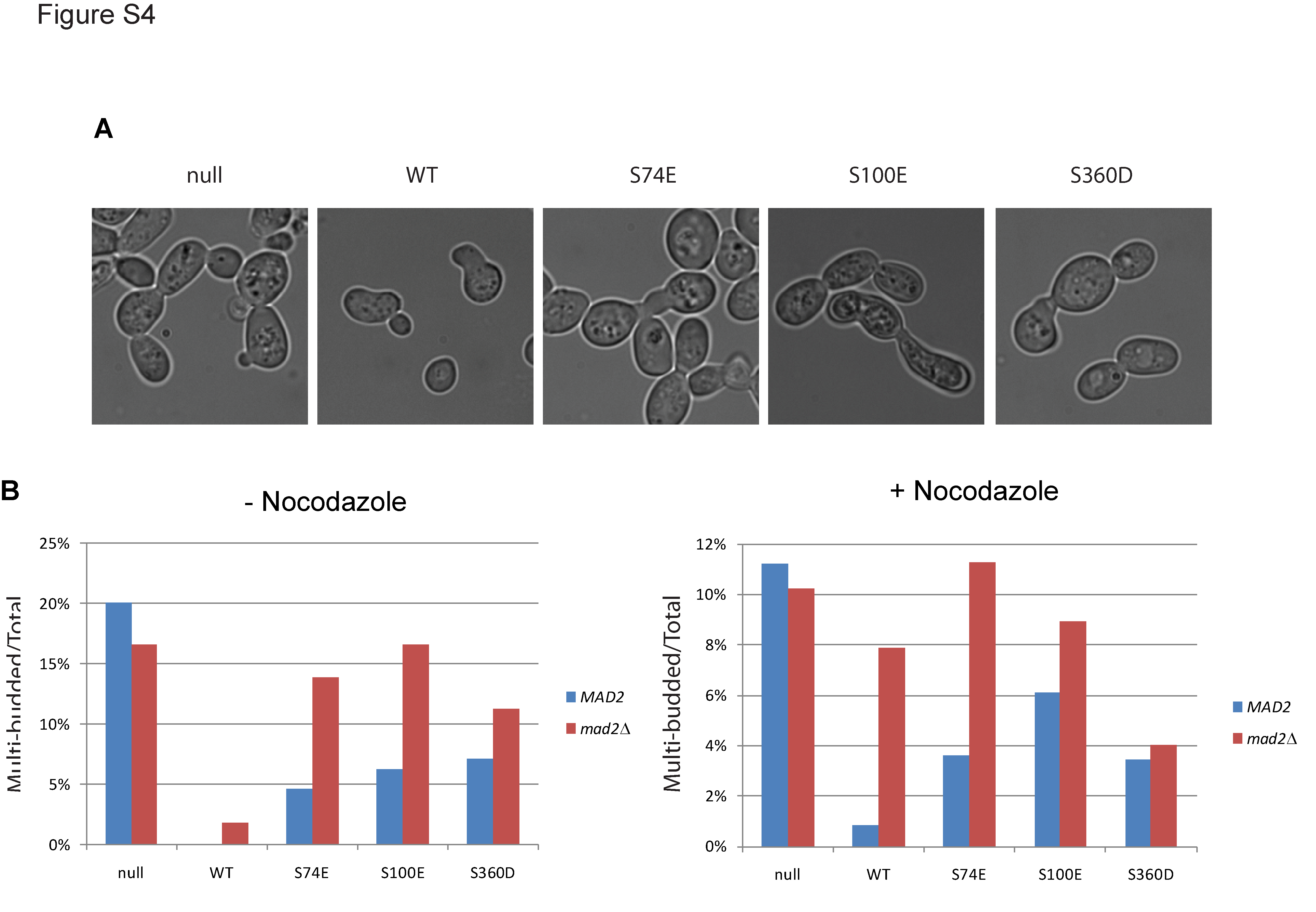

Supplement: Figure S4 — Multi-budded cells emerged when spindle assembly check point gene MAD2 was deleted. (A) Micrographs of TUB4-AID mad2Δ cells with empty vector (“null”), TUB4, tub4-S74E, tub4-S100E or tub4-S360D were arrested in G1-phase with α-factor. IAA was added 30 min before G1 release by washing cells with YPAD containing IAA (G1 release t = 0). Cells were fixed with 70% ethanol after 3 hours from releasing. (B) The percentage of multi-budded cells in each TUB4-AID MAD2 cells (blue bar) and TUB4-AID mad2Δ cells (red bar) with/without nocodazole treatment. (TIF) [file pone.0019700.s004.tif]

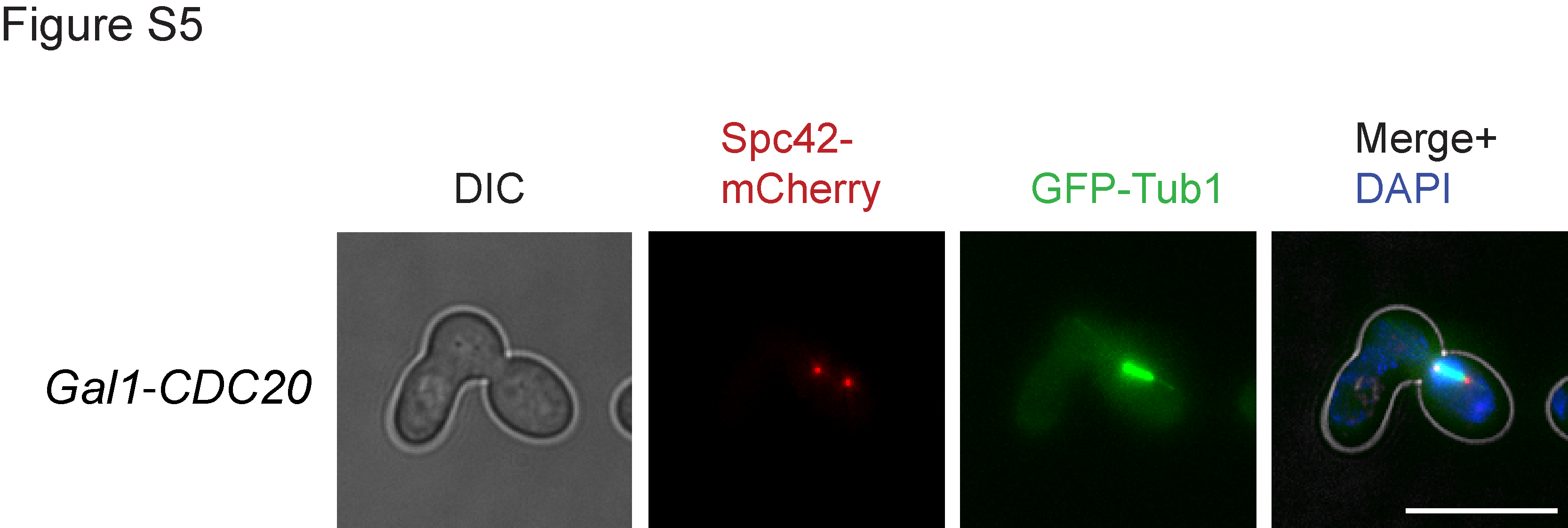

Supplement: Figure S5 — Spindle length of metaphase-arrested Gal1- CDC20 cells. Micrographs of Gal1-CDC20 SPC42-mCherry GFP-TUB1 cells grown for 90 min in glucose medium to repress expression of Gal1-CDC20 and to induce arrest of cells in metaphase. Genomic DNA was stained with DAPI. Scale bar: 10 µm. (TIF) [file pone.0019700.s005.tif]

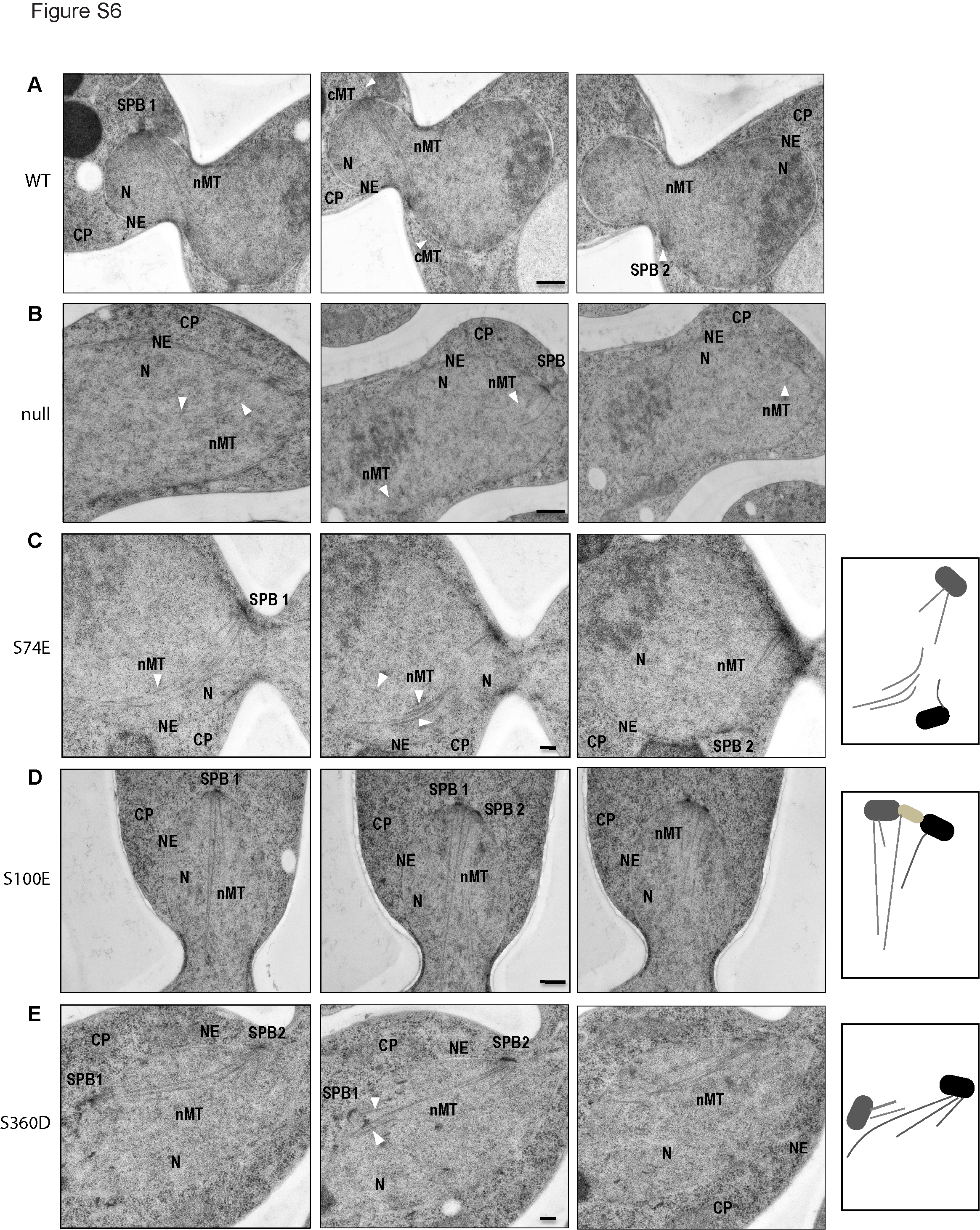

Supplement: Figure S6 — EM images of microtubule phenotypes of tub4-S74E , tub4-S100E and tub4-S360D cells. (A-E) TUB4-AID cells with the wild type TUB4 (“WT”) (A), empty plasmid (“null”; B), tub4-S74E (C), tub-S100E (D) or tub4-S360D (E) were synchronized with α-factor and treated with IAA as described in Figure 4A. 45 or 75 min after the G1 release cells were prepared for thin serial sectioning and observed under electron microscopy as described in Materials and Methods. (A-D) Shown are three consecutive serial sections. The white arrows indicate the position of defective nuclear microtubules. Abbreviations: CP, cytoplasm; cMT, cytoplasmic microtubules; N, nucleus; NE, nuclear envelope; nMT, nuclear microtubules; SPB, spindle pole body. Scale bar: 200 nm. (TIF) [file pone.0019700.s006.tif]

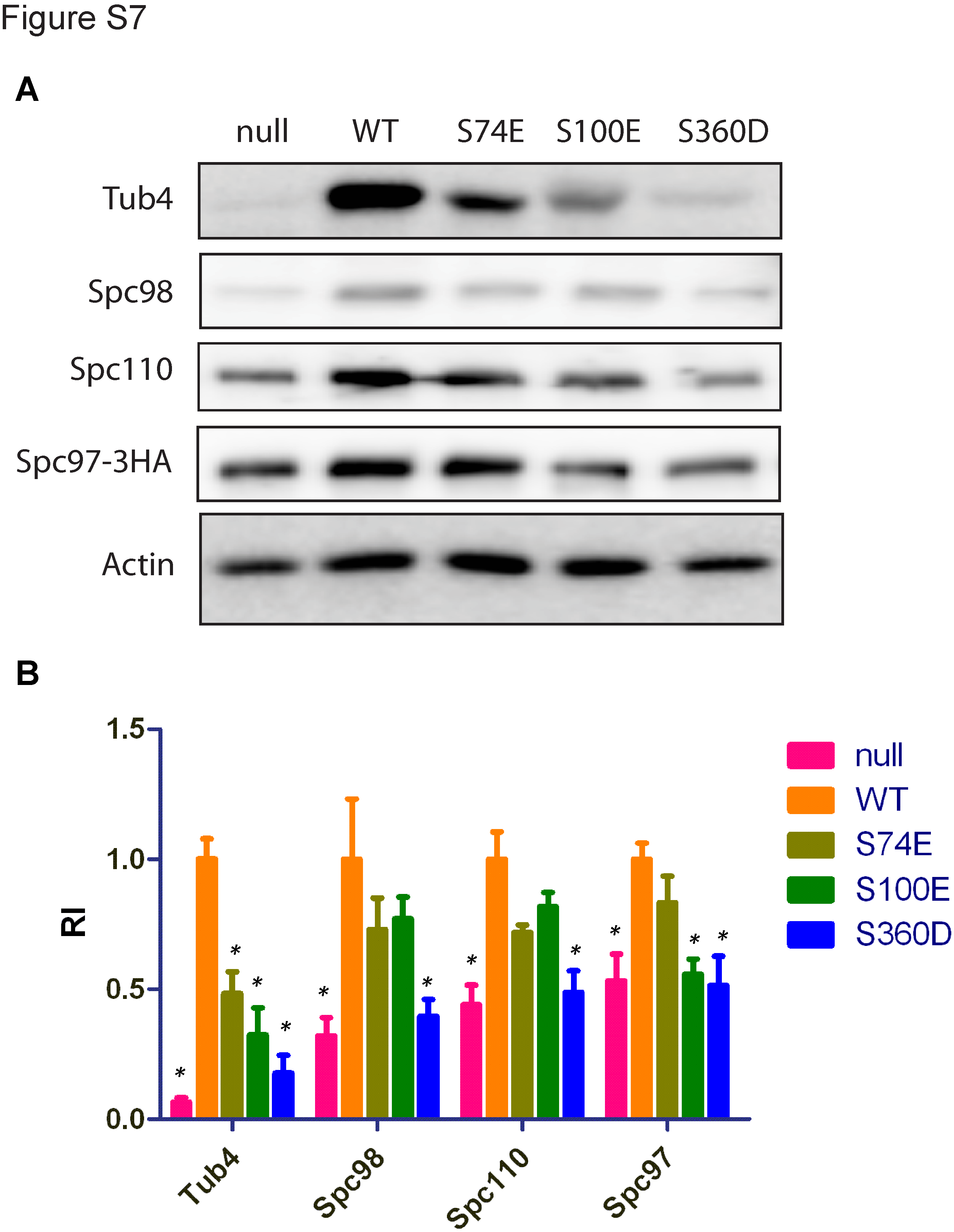

Supplement: Figure S7 — Western blot of TCA cell extracts. (A) Asynchronous TUB4-AID cells were grown in YPAD at 30°C to mid log-phase. Depletion of Tub4-AID was achieved by incubation of cells with 0.5 mM IAA for 2 h. After TCA extraction total cell lysates were analyzed by SDS-PAGE and immunoblotting with anti-Tub4, anti-Spc98, anti-Spc110 and anti-HA (Spc97-3HA blot) antibodies. Anti-actin antibodies were used to normalize loading. (B) Protein levels of cell extracts from (A) were quantified and normalized as described in Figure 6. Significance of the difference between wild-type and mutants at p<0.05 was determined by one-way ANOVA and is indicated by an asterisk. (TIF) [file pone.0019700.s007.tif]
